# Characterization of dipyridamole as a novel ferroptosis inhibitor and its therapeutic potential in acute respiratory distress syndrome management

**DOI:** 10.7150/thno.102318

**Published:** 2024-10-21

**Authors:** Xu Chen, Jiapan Shen, Xueqin Jiang, Min Pan, Shuang Chang, Juanjuan Li, Lei Wang, Manli Miao, Xiaoxia Feng, Ling Zhang, Guoqing Shu, Wenjian Liu, Fangzhou Xu, Wentao Zhang, Zhao Ding, Huaiyuan Zong, Weiwei Liu, Dapeng Li, Biao Chen, Min Shao, Guanghe Fei, Xiaojun Zha, Xiaoyun Fan

**Affiliations:** 1 Department of Geriatric Respiratory and Critical Care Medicine, The First Affiliated Hospital of Anhui Medical University, Hefei, Anhui, China.; 2 Department of Biochemistry & Molecular Biology, School of Basic Medicine, Anhui Medical University, Hefei, Anhui, China.; 3 Anhui Geriatric Institute, Hefei, Anhui, China.; 4 Department of Respiratory and Critical Care Medicine, The First Affiliated Hospital of Anhui Medical University, Hefei, Anhui, China.; 5 Key Laboratory of Respiratory Diseases Research and Medical Transformation of Anhui Province, Hefei, Anhui, China.; 6 Department of Thoracic Surgery, The First Affiliated Hospital of Anhui Medical University, Hefei, Anhui, China.; 7 Information Materials and Intelligent Sensing Laboratory of Anhui Province, Institutes of Physical Science and Information Technology, Anhui University, Hefei, Anhui, China.; 8 Department of Critical Care Medicine, The First Affiliated Hospital of Anhui Medical University, Hefei, Anhui, China.; Xu Chen, Jiapan Shen, and Xueqin Jiang share the first author position in this work.

**Keywords:** ferroptosis, dipyridamole, acute respiratory distress syndrome, heme oxygenase 1, cAMP responsive element binding protein 1

## Abstract

**Rationale**: Ferroptosis in lung epithelium and endothelium contributes to the pathogenesis of acute respiratory distress syndrome (ARDS), a critical and often fatal condition marked by acute inflammation and elevated pulmonary vascular permeability. Despite this, there are currently no FDA-approved therapeutics specifically targeting ferroptosis for ARDS management.

**Methods**: A screening of 259 FDA-approved drugs was conducted to identify an effective ferroptosis inhibitor in pulmonary epithelial and endothelial cells. The anti-ferroptotic and therapeutic efficacy of this screened drug was rigorously evaluated using two distinct ARDS mouse models (LPS-induced acute lung injury and CLP-induced sepsis) and human airway organoids (hAOs). The regulatory mechanism of this drug on ferroptosis inhibition was investigated via RNA-sequencing, qRT-PCR, western blotting, IF, luciferase reporter assay, chromatin immunoprecipitation assay, limited proteolysis-mass spectrometry assay, cellular thermal shift assay, and drug affinity responsive target stability assay. Furthermore, a proof-of-concept clinical trial was conducted, wherein ARDS patients were administered with the drug as adjunctive therapy.

**Results**: Dipyridamole (DIPY) was identified as a potent inhibitor of ferroptosis in pulmonary epithelial and endothelial cells. DIPY effectively mitigated ferroptosis and pulmonary damage in both mouse models and hAOs, primarily by downregulating heme oxygenase 1 (HMOX1). The transcription factor cAMP responsive element binding protein 1 (CREB1) was identified as a key transactivator of HMOX1, which DIPY effectively downregulated. Mechanistically, DIPY binds to and activates superoxide dismutase 1 (SOD1), which in turn inhibits the CREB1/HMOX1 pathway, thereby suppressing ferroptosis. Notably, the clinical trial further corroborated the therapeutic potential of DIPY in ARDS patients, demonstrating improved outcomes with DIPY adjunctive therapy.

**Conclusions**: These findings provide compelling evidence that DIPY inhibits ferroptosis in pulmonary epithelial and endothelial cells by modulating the SOD1/CREB1/HMOX1 signaling axis and suggest DIPY as a promising therapeutic strategy for ARDS treatment.

## Introduction

Acute Respiratory Distress Syndrome (ARDS) is a severe inflammatory condition of the lungs characterized by rapid onset of widespread inflammation, increased pulmonary vascular permeability, and severe hypoxemia 1. High incidence, morbidity, and high medical expenditure make ARDS one of the most prevalent and important complications in the intensive care unit 1. Pulmonary local injury and systematic inflammation are the two major causes of ARDS 2. Despite advances, ARDS treatment is limited by the lack of effective pharmacological therapies and the potential for mechanical ventilation to exacerbate lung injury, highlighting the need for more effective approaches 3. Pulmonary epithelial and endothelial cells are crucial for maintaining lung function and the integrity of the alveolar-capillary barrier. Injury to this barrier is characteristic of ARDS, impairing surfactant production and fluid clearance while promoting vascular leakage. This leads to increased lung inflammation and worsened hypoxemia. Therefore, targeting the damage to pulmonary epithelial and endothelial cells holds promise as a breakthrough approach in ARDS treatment.

Ferroptosis is a non-apoptotic cell death mechanism characterized by iron-dependent membrane lipid peroxidation 4. The core signaling pathway of ferroptosis revolves around iron metabolism, the glutathione (GSH)/glutathione peroxidase 4 (GPX4) axis, and lipid peroxidation 5. Free intracellular iron reacts with oxygen and polyunsaturated fatty acid (PUFA) to generate high levels of membrane lipid peroxides, which is one of the most obvious characteristics of ferroptosis compared to other forms of cell death 6. The accumulation of these membrane lipid peroxides at high levels can be lethal to the cell 7. Morphologically, shrunken mitochondria with increased membrane density and reduced mitochondrial cristae of cells undergoing ferroptosis are another hallmark of ferroptosis 8. Ferroptosis can be induced by compounds such as RAS-selective lethal 3 (RSL3), a GPX4 inhibitor, while antioxidants like Ferrostatin-1 and Liproxstatin-1 can effectively suppress it 4, 9. Increasing research has indicated that dysregulated iron metabolism is reported in ARDS patients 8, 10, 11. Specifically, total and non-heme iron levels were higher in the plasma and bronchoalveolar lavage fluid (BALF) of ARDS patients compared to healthy controls 10, 11. Moreover, lipid hydroperoxides were found in the pulmonary edema fluid of ARDS patients 12. Preclinical studies have demonstrated that iron-dependent ferroptotic damage to lung epithelial and endothelial cells exacerbates ARDS. Additionally, the inhibition of ferroptosis has been shown to mitigate lung injury in experimental models of ARDS 13, 14. However, there are no Food and Drug Administration (FDA)-approved drugs specifically targeting ferroptosis for the clinical treatment of ARDS.

In this study, we identified Dipyridamole (DIPY) as a powerful inhibitor of ferroptosis in lung epithelium and endothelium by screening a library of FDA-approved drugs. We further confirmed the anti-ferroptotic and therapeutic effects of DIPY in two ARDS mice models and human airway organoids (hAOs). Notably, a proof-of-concept trial demonstrated that DIPY adjunctive therapy significantly mitigated ferroptosis and provided clinical benefit to patients with ARDS. Moreover, we uncovered that DIPY suppresses ferroptosis by regulating the SOD1/CREB1/HMOX1 pathway.

## Results

### The FDA-approved drug screening identifies DIPY as a new ferroptosis inhibitor in lung epithelial and endothelial cells

**Figure 1:**
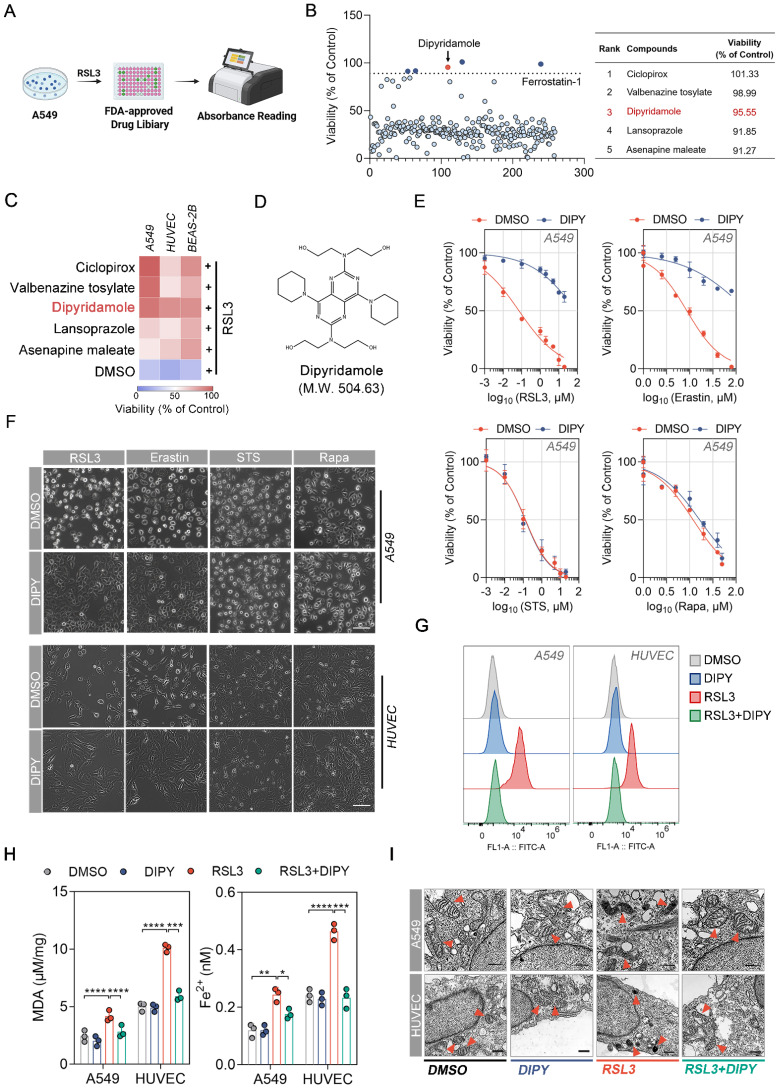
** The FDA-approved drug screening identifies DIPY as a new ferroptosis inhibitor in lung epithelial and endothelial cells.** (**A**) Schematic diagram of drug screening. A549 cells were treated with RSL3 (5 µM) and 259 FDA-approved drugs for 8 hours, followed by CCK-8 assays. (**B**) Scatter plot showing the viability of A549 cells treated with various compounds from the FDA-approved drug library in the presence of RSL3 normalized to control (left panel). The top five candidate compounds with the highest viability percentages are identified and listed in the table (right panel). (**C**) A549, HUVEC, and BEAS-2B cells were treated with Ciclopirox, Valbenazine tosylate, DIPY, Lansoprazole, Asenapine maleate, or DMSO, together with RSL3 (5 µM) for 8 hours. Cell viability was assessed and the results were presented as a heat map. (**D**) The chemical structure of DIPY is shown. (**E** and **F**) A549 and HUVEC cells were treated with RSL3, Erastin, STS, or Rapa, together with DIPY (10 µM for A549; 5 µM for HUVEC) or DMSO for indicated times (RSL3 and Erastin for 8 hours; STS for 6 hours; Rapa for 48 hours). The cell viability of A549 cells was measured (**E**). Representative phase-contrast images of cells were captured (**F**). Scale bar, 100 μm. (**G**-**I**) A549 cells were treated with RSL3 (5 µM), DIPY (10 µM), and RSL3 (5 µM) plus DIPY (10 µM) for 8 hours. HUVEC cells were treated with RSL3 (0.25 µM), DIPY (5 µM), and RSL3 (0.25 µM) plus DIPY (5 µM) for 8 hours. L-ROS levels were assessed using C11-BODIPY (**G**). MDA contents and Fe^2+^ levels were measured (**H**). Representative TEM images of cells were taken, with red arrows indicating mitochondria (**I**). Scale bar, 500 nm. Results are shown as mean ± SD from 3 independent experiments. Statistical significance is indicated as *P < 0.05; **P < 0.01; ***P < 0.001; ****P < 0.0001.

To identify potent and specific inhibitors of ferroptosis in lung epithelial and endothelial cells, we screened a library of 259 FDA-approved drugs for their ability to inhibit ferroptosis caused by RSL3 (a recognized ferroptosis inducer) in human alveolar epithelial cell line A549 (Figure 1A) 15. Cells were treated with a final concentration of 10 μM of drugs plus RSL3 for 8 h and the cell viability was determined by CCK-8 assays. By using Ferrostatin-1 (a common ferroptosis inhibitor) as a positive criterion, 5 compounds that significantly inhibited RSL3-induced A549 cell death are screened and listed in the right panel of Figure 1B. Furthermore, the antiferroptoic roles of these 5 drugs were evaluated in A549, human umbilical vein endothelial cells (HUVEC), and human bronchial epithelial cells (BEAS-2B). The results demonstrated that only Dipyridamole (DIPY) significantly blocked RSL3-induced cell death in all three cell lines (Figure 1C-D).

To confirm that DIPY specifically inhibits ferroptosis, but not other types of cell death such as apoptosis and autophagy, we performed CCK-8 assays to evaluate the effect of DIPY on the lethality triggered by various compounds in A549, HUVEC, and BEAS-2B cells. As shown in Figure 1E-F and Figure S1A-C, DIPY specifically inhibited RSL3- and Erastin-induced cell death, but not cell death induced by other lethal compounds such as apoptosis-inducing agents Staurosporine (STS), and autophagy-inducing agents Rapamycin (Rapa).

Given that ferroptosis is characterized by elevated intracellular levels of iron and lipid reactive oxygen species (L-ROS), as well as the accumulation of lipid peroxides during cell death 4, we detected the L-ROS levels, lipid peroxides, and Fe^2+^ levels in A549, HUVEC, and BEAS-2B cells after being treated with RSL3 and DIPY. As shown in Figure 1G and Figure S1D, using BODIPY-C11 (a lipid-soluble ratiometric fluorescent indicator of lipid peroxidation), DIPY prevented RSL3-induced accumulation of L-ROS in all these cells. Also, DIPY attenuated malondialdehyde (MDA, one of the final products of lipid peroxidation) contents and iron levels elevated by RSL3 (Figure 1H and Figure S1E). Moreover, using transmission electron microscopy (TEM), we observed that shrunken mitochondria and increased membrane density induced by RSL3 were recovered by the treatment of DIPY in these cells (Figure 1I and Figure S1F). We also assessed the expression levels of several ferroptosis-related markers, including ACSL4, SLC7A11, GPX4, and FTH1. Interestingly, the expression of these ferroptosis-associated proteins exhibited only a minimal alteration in response to DIPY treatment (Figure S1G). The above results indicated that DIPY significantly protects lung epithelial and endothelial cells against ferroptosis.

### DIPY inhibits ferroptosis in mice models of ARDS

**Figure 2:**
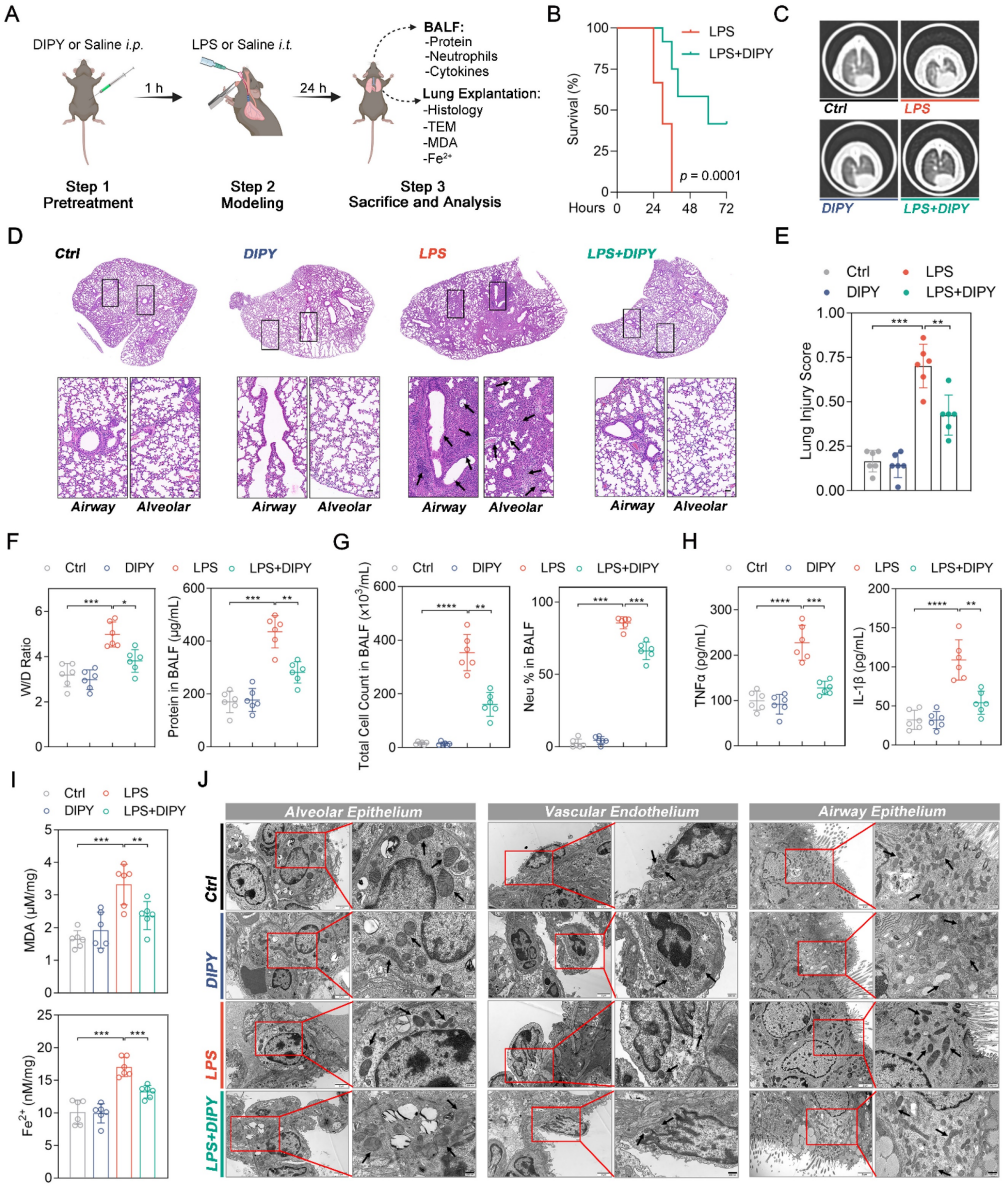
** DIPY inhibits ferroptosis in LPS-induced ARDS mouse model.** (**A**) Scheme of the experimental procedure for the LPS-induced ARDS mouse model. DIPY treatment (10 mg/kg, intraperitoneal) was administered 1 hour before the LPS challenge (low dose: 2.5 mg/kg; lethal dose: 20 mg/kg). After 24 hours, the BALF and the lungs of the mice (treated with low-dose LPS) were collected and analyzed. (**B**) Percent survival after administering lethal doses of LPS instillation is shown (n = 12 per group). (**C-J**) Low-dose LPS-challenged ARDS mice were pretreated with or without DIPY (n = 6 per group). Representative CT images of mouse lungs (**C**). Representative images of H&E-stained lung sections, with black arrows indicating infiltrating inflammatory cells, interstitial edema, and alveolar wall thickening (**D**). Scale bar, 50 μm. Lung injury scores (**E**). Measurements of lung wet/dry ratio and total protein in BALF (**F**). Total cell counts and neutrophil percentage in BALF (**G**). TNF-α and IL-1β expressions in BALF were measured by ELISA (**H**). MDA contents (upper panel) and Fe^2+^ levels (lower panel) in mouse lung tissues (**I**). Representative TEM images illustrating mitochondria in the alveolar epithelium, vascular endothelium, and airway epithelium, with black arrows indicating mitochondria (**J**). Scale bar, 500 nm. Results are shown as mean ± SD. Statistical significance is indicated as *P < 0.05; **P < 0.01; ***P < 0.001; ****P < 0.0001.

Next, LPS-induced acute lung injury (ALI) mice were employed to validate the *in vivo* anti-ferroptotic effects of DIPY in ARDS. As shown in Figure 2A, pretreatment with DIPY (10 mg/kg, *ip.*) or Saline for 1 h, followed by exposure to intratracheal LPS (low dose: 2.5 mg/kg; lethal dose: 20 mg/kg) or Saline instillation for the indicated time, the mice (low-dose LPS treated) were then sacrificed and the BALF and lung tissues were collected. Chest computed tomography (CT) was performed before the mice were killed. As shown in Figure 2B, we observed 100% mortality in mice challenged with lethal-dose LPS, whereas LPS-challenged mice receiving DIPY showed markedly reduced mortality. CT imaging revealed a marked increase in pulmonary exudative lesions following low-dose LPS administration, which was significantly mitigated by DIPY pretreatment (Figure 2C). Histological assessment of lung sections showed that DIPY markedly prevented LPS-induced damage to pulmonary epithelial cells and vascular endothelium, evidenced by the reduced interstitial edema and decreased alveolar wall thickening (Figure 2D-E). To further investigate the effects of DIPY on pulmonary edema and the inflammatory response in LPS-challenged mice, lung wet/dry (W/D) ratio, total protein, total cell counts, neutrophil percentage, TNF-α, and IL-1β in BALF were determined. As shown in Figure 2F-H, DIPY strongly inhibited lung wet/dry ratio and total protein in BALF, accompanied by decreased total cell counts, neutrophil infiltration, and inflammatory factors. As expected, pretreatment with DIPY reduced MDA contents and iron levels induced by LPS in lung tissues (Figure 2I). TEM analyses showed that the mitochondria were severely damaged with increased membrane density in alveolar epithelium, vascular endothelium, and airway epithelium, after LPS intratracheal instillation, which was rescued by DIPY pretreatment (Figure 2J). DIPY treatment resulted in a modest upregulation of SLC7A11, GPX4, and FTH1, whereas the expression of ACSL4 was slightly downregulated compared to the ARDS models (Figure S2).

The above findings were further validated in a murine cecal ligation and puncture (CLP) model of polymicrobial sepsis (Figure S3A). As shown in Figure S3B-C, DIPY significantly prevented CLP-induced death and decreased the clinical score of mice. Consistent with the LPS-induced ARDS model, the increased lung injury, inflammation, and ferroptosis in the sepsis group were significantly reversed by DIPY pretreatment (Figure S3D-I). Taken together, these findings suggested that DIPY might prevent ARDS by inhibiting ferroptosis.

### DIPY inhibited ferroptosis in hAOs

**Figure 3:**
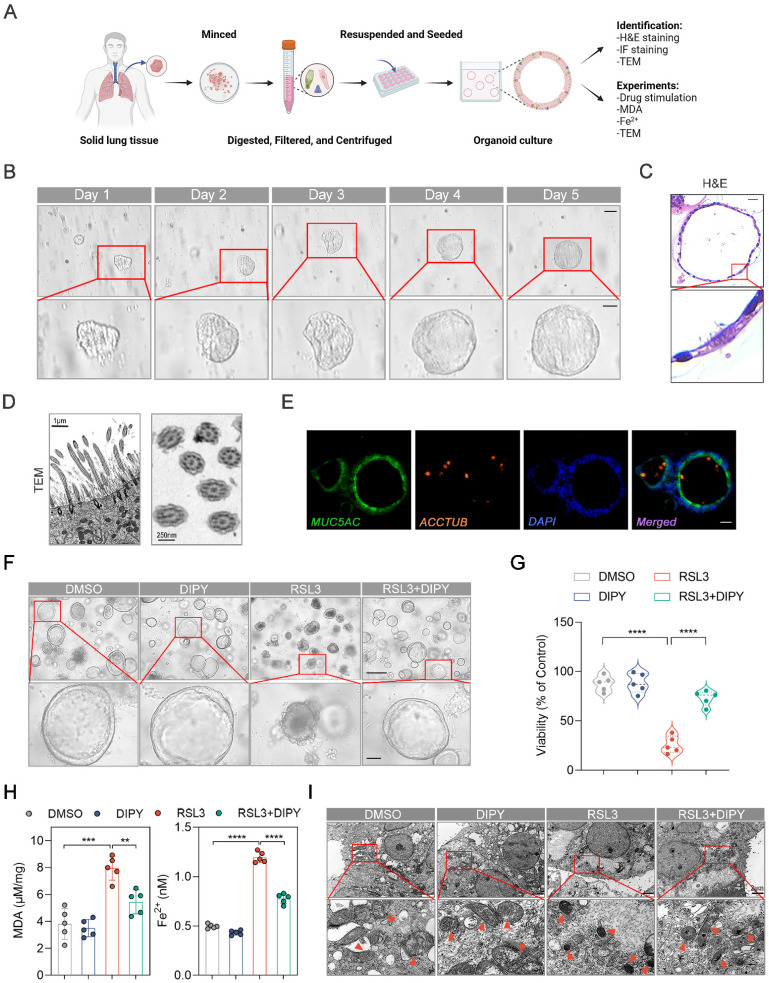
** DIPY inhibits ferroptosis in hAOs.** (**A**) Workflow for establishing and identifying hAOs from solid lung tissue. (**B**) Brightfield images of cultured hAOs derived from patients. Scale bar, 100 μm (upper panel) and 50 μm (lower panel). (**C**) Representative images of H&E-stained sections of hAOs, showing basal and multi-ciliated cells. Scale bar, 20 μm. (**D**) TEM images reveal detailed longitudinal and cross-sections of the cilia in the organoids, demonstrating the typical microtubule arrangement. Scale bar, 1 μm (left panel) and 250 nm (right panel). (**E**) Identification of the mucosecretory and ciliated cells in the hAOs by IF assays. Scale bar, 10 μm. (**F-I**) The hAOs were treated with RSL3 (30 μM), DIPY (20 μM), and RSL3 (30 μM) with DIPY (20 μM) for 24 hours (n = 5 per group). Morphological changes of hAOs (**F**). Scale bar, 250 μm (upper panel) and 50 μm (lower panel). Assessment of organoid viability (**G**). Measurement of MDA contents and Fe^2+^ levels (**H**). TEM images illustrate representative mitochondria (black arrows) in the hAOs (**I**). Scale bar, 2 μm. Results are shown as mean ± SD. Statistical significance is indicated as **P < 0.01; ***P < 0.001; ****P < 0.0001.

Organoids, self-organizing 3D structures derived from stem cells or tissues, remain highly reductionist versions of real tissues and bridge the gap between animal models and clinical studies 16-18. We have generated human airway organoids (hAOs), using distal normal lung tissue from non-small cell lung cancer (NSCLC) patients undergoing general surgery (Figure 3A-B). As shown in Movie S1, the dynamic moving cilia in the hAOs were captured. Hematoxylin/eosin (H&E) staining showed the basal and multi-ciliated cells of the organoids (Figure 3C). Also, the longitudinal section and cross-section of cilia from our hAOs exhibited a healthy, well-ciliated strip of respiratory epithelium by using TEM (Figure 3D). Moreover, cell types of the organoids were determined by immunofluorescence (IF) using the indicated markers: MUC5AC (green) for mucosecretory cells and ACCTUB (red) for ciliated cells (Figure 3E). Therefore, we have successfully established hAOs.

To evaluate the anti-ferroptosis potential of DIPY in organoids, we treated hAOs with RSL3 and DIPY. As shown in Figure S4A, DIPY was efficiently delivered into airway epithelial cells of hAOs. The hAOs displayed obvious ferroptosis morphological characterizations (shrinkage, concentration, and disintegration) after RSL3-challenged, while DIPY treatment reversed these morphological changes (Figure 3F). The CellTiter-Glo^®^ 3D cell viability assay demonstrated that DIPY recovered the viability of organoids suppressed by ferroptosis inducer (Figure 3G). Furthermore, the cellular MDA and Fe^2+^ were evaluated to show that DIPY could protect the hAOs from lipid peroxidation damage and ferroptosis (Figure 3H). Moreover, TEM revealed that damaged mitochondria in RSL3-stimulated hAOs were alleviated by DIPY treatment (Figure 3I). Similar to RSL3, LPS-induced lung epithelial injury and ferroptosis in hAOs were also significantly mitigated in response to DIPY treatment (Figure S4B-E). Altogether, these data indicated that DIPY inhibits ferroptosis in hAOs.

### HMOX1 is a target of DIPY

**Figure 4:**
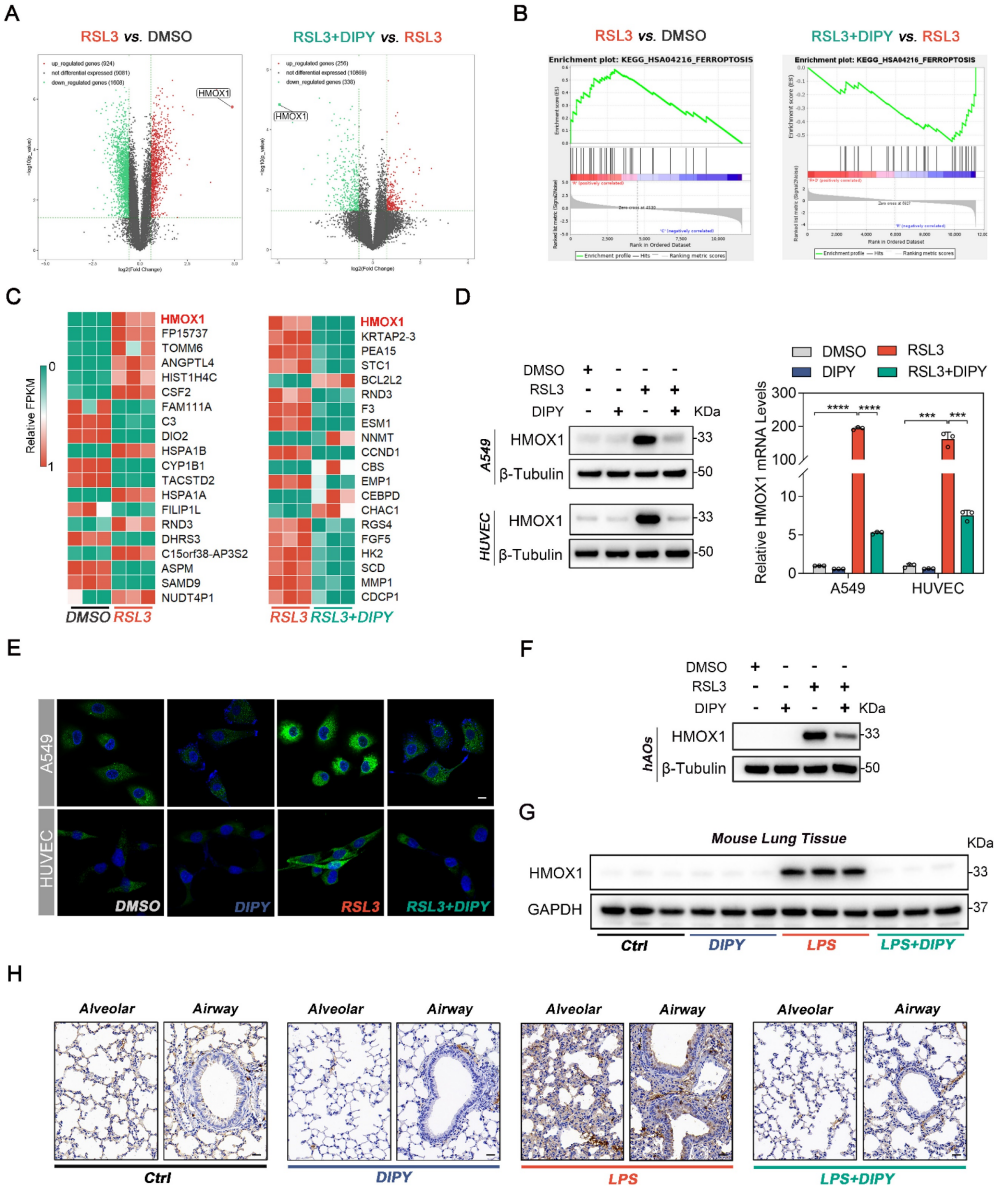
** HMOX1 is a target of DIPY.** (**A**) Volcano plots of RNA-seq analysis (n = 3 per group). (**B**) GSEA analysis of RNA-seq data. (**C**) Heat maps of the top 20 DEGs in both comparative groups. (**D** and** E**) A549 and HUVEC cells were treated with RSL3, DIPY, and RSL3 with DIPY for 8 hours as shown in Figure 1 (n = 3 per group). The expression of HMOX1 was detected by western blot (left panel of **D**), qRT-PCR (right panel of **D**), and IF staining (**E**). Scale bar, 10 μm. (**F**) hAOs were treated with RSL3, DIPY, and RSL3 with DIPY for 24 hours as shown in Figure 3, and the protein levels of HMOX1 were analyzed by western blot (n = 5 per group). (**G** and** H**) The expression of HMOX1 in LPS-induced ALI mouse models with or without DIPY treatment was detected by western blot (**G**) and IHC (**H**) assays (n = 6 per group). Scale bar, 50 μm. Results are shown as mean ± SD of 3-6 independent experiments. Statistical significance is indicated as ***P < 0.001; ****P < 0.0001.

To explore the molecular mechanism of DIPY against ferroptosis, we performed high-throughput RNA sequencing (RNA-Seq) analysis by utilizing RSL3- or RSL3 plus DIPY-treated A549 cells. The result showed that 2532 different expression genes (DEGs, 924 upregulated and 1608 downregulated genes) of RSL3-treated *vs.* DMSO-treated A549 cells, and 594 DEGs (256 upregulated and 338 downregulated genes) of cells co-incubated with DIPY and RSL3 *vs.* RSL3-treated cells were identified (Figure 4A). Gene set enrichment analysis (GSEA) showed ferroptosis genes were significantly enriched in RSL3-treated cells compared to control cells (the left panel of Figure 4B). In contrast, compared to the RSL3 treatment group, the RSL3 plus DIPY-treated group negatively correlated with ferroptosis genes (the right panel of Figure 4B). Furthermore, we list the top 20 DEGs of these two comparative groups (Figure 4C). Interestingly, among these genes, heme oxygenase 1 (HMOX1) ranks first both in these two comparative groups. Subsequently, immunoblotting, quantitative real-time PCR (qRT-PCR), and IF assays verified that RSL3 strongly upregulated HMOX1 in lung epithelial and endothelial cells, while its expression was significantly suppressed by DIPY (Figure 4D-E and Figure S5A-C). Furthermore, we confirmed that DIPY treatment attenuated the upregulated HMOX1 in RSL3- or LPS-challenged hAOs (Figure 4F and Figure S5D). In addition, the increased HMOX1 expression in the lung epithelial and endothelial cells was reversed by DIPY treatment in both LPS-induced ALI and CLP-induced sepsis mice (Figure 4G-H and Figure S5E-F). Therefore, HMOX1 is a downstream target of DIPY and DIPY may prevent ferroptosis via HMOX1 inhibition.

### DIPY inhibits ferroptosis through suppression of HMOX1

**Figure 5:**
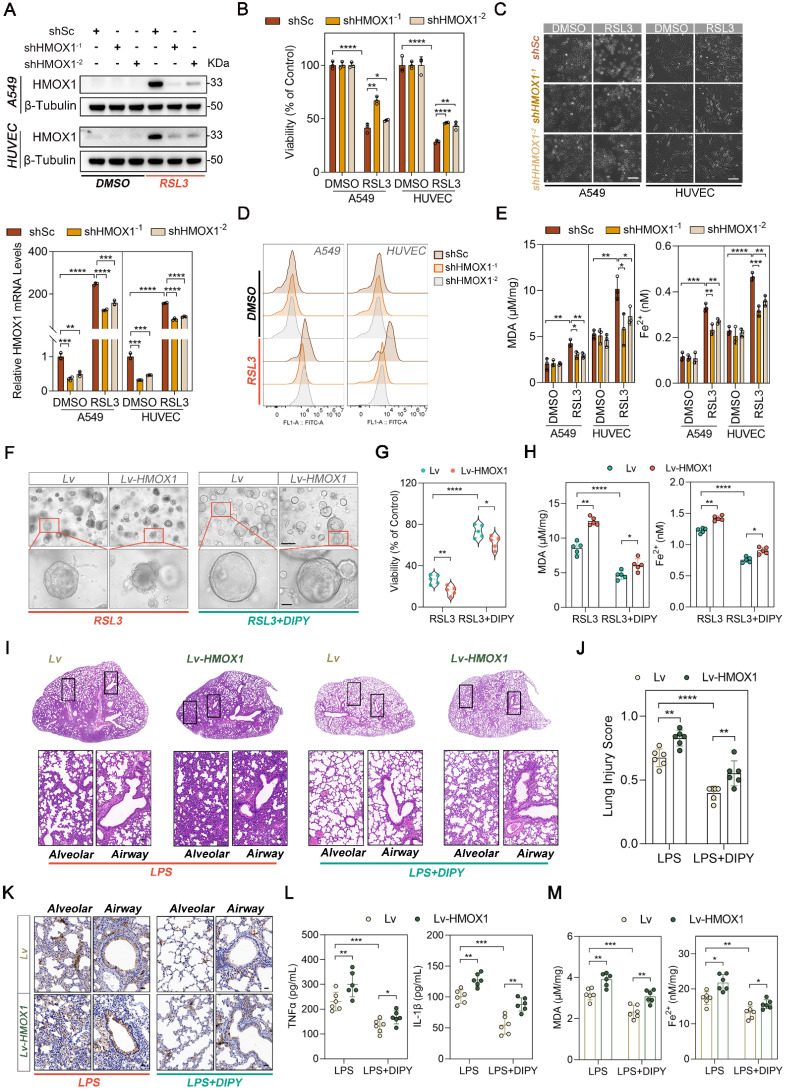
** DIPY inhibits ferroptosis through suppression of HMOX1.** (**A-E**) A549 and HUVEC cells were infected with lentivirus expressing shRNAs against HMOX1 (shHMOX1^-1^ and shHMOX1^-2^) or a scrambled sequence (shSc). These cells were stimulated with RSL3 (5 μM for A549; 0.25 μM for HUVEC) for 8 hours (n = 3 per group). HMOX1 expression was analyzed by western blot (upper panel of **A**) and qRT-PCR (lower panel of **A**). Cell viability assessment (**B**). Representative phase-contrast images of cells (**C**). Scale bar, 100 μm. L-ROS levels measurement (**D**). MDA contents and Fe^2+^ levels (**E**). (**F-H**) The hAOs were infected with lentivirus harboring a vector encoding HMOX1 (Lv-HMOX1) or the empty vector (Lv). These hAOs were treated by RSL3 (30 μM) with or without DIPY (20 μM; n = 5 per group). Representative phase-contrast images of hAOs (**F**). Scale bar, 250 μm (upper panel) and 50 μm (lower panel). Viability assessment of organoids (**G**). MDA contents and Fe^2+^ levels (**H**). (**I-M**) C57/BL6 mice were administered HMOX1-overexpressed lentivirus (Lv-HMOX1) or the empty vector (Lv) via intratracheal instillation for 7 days. On day 8, the indicated mice were challenged with LPS (2.5 mg/kg) with or without DIPY pretreatment (n = 6 per group). Representative images of H&E-stained lung sections (**I**). Scale bar, 50 μm. Lung injury scores (**J**). IHC staining of HMOX1 in mice lung sections (**K**). Scale bar, 50 μm. ELISA analysis of TNF-α and IL-1β expressions in BALF (**L**). MDA and Fe^2+^ levels in lung tissues (**M**). Results are shown as mean ± SD of 3-6 independent experiments. Statistical significance is indicated as *P < 0.05; **P < 0.01; ***P < 0.001; ****P < 0.0001.

We next investigate whether HMOX1 mediated the antiferroptic role of DIPY in epithelial and endothelial cells. Firstly, we inhibited HMOX1 by using zinc protoporphyrin (ZnPP, a competitive inhibitor of HMOX1) in RSL3-treated A549 or HUVEC cells (Figure S6A). The CCK-8 assay and phase-contrast images of cells indicated that RSL3-induced cell death was attenuated significantly by ZnPP (Figure S6B-C). In addition, ZnPP treatment partially rescued the L-ROS, MDA, and Fe^2+^ elevated by RSL3 (Figure S6D-E). Therefore, HMOX1 inhibition prevented cell sensitivity to ferroptosis like DIPY. Secondly, we established stable HMOX1 knockdown A549 and HUVEC cells to verify the function of HMOX1 in ferroptosis. The efficacy of HMOX1 knockdown was confirmed by western blot and qRT-PCR (Figure 5A). As shown in Figure 5B-E, knockdown of HMOX1 significantly suppressed RSL3-induced cell death, and prevented RSL3-induced accumulation of lipid peroxidation and iron levels compared with the control in A549 and HUVEC cells. In contrast, HMOX1 was ectopically overexpressed in A549 and HUVEC cells (Figure S6F). Contrary to the HMOX1 inhibition or knockdown, HMOX1 overexpression attenuated DIPY-induced cell death and ferroptosis inhibition (Figure S6G-J).

To further investigate the role of HMOX1 on ferroptosis, we generated HMOX1-overexpressed hAOs. As depicted in Figure 5F-G, RSL3-induced organoid disorganization and decreased organoid viability were rescued by DIPY, and this effect was attenuated by HMOX1 overexpression. What's more, the overexpression of HMOX1 weakened DIPY-mediated the decrease in lipid peroxidation levels and intracellular iron accumulation in hAOs (Figure 5H).

In addition, we investigated whether HMOX1 overexpression affects the anti-inflammation and ferroptosis resistance effects of DIPY in LPS-induced ARDS mice. As shown in Figure 5I-L and Figure S6K, intratracheal instillation of HMOX1-overexpressed lentivirus reduced the therapeutic effectiveness of DIPY in ARDS mice, as evidenced by exacerbated alveolar septa thickening, aggravated infiltration of inflammatory cells into the bronchioles, vascular bed, and lung parenchyma, and increased inflammatory factors (TNF-α and IL-1β) in BALF after HMOX1 overexpression. Strikingly, overexpression of HMOX1 significantly diminished the inhibitory effects of DIPY on MDA and Fe^2+^ levels in lung tissues of ARDS mice (Figure 5M). Taken together, these data indicated that DIPY suppresses ARDS and ferroptosis through inhibition of HMOX1.

### DIPY suppresses HMOX1 through the deactivation of CREB1

**Figure 6:**
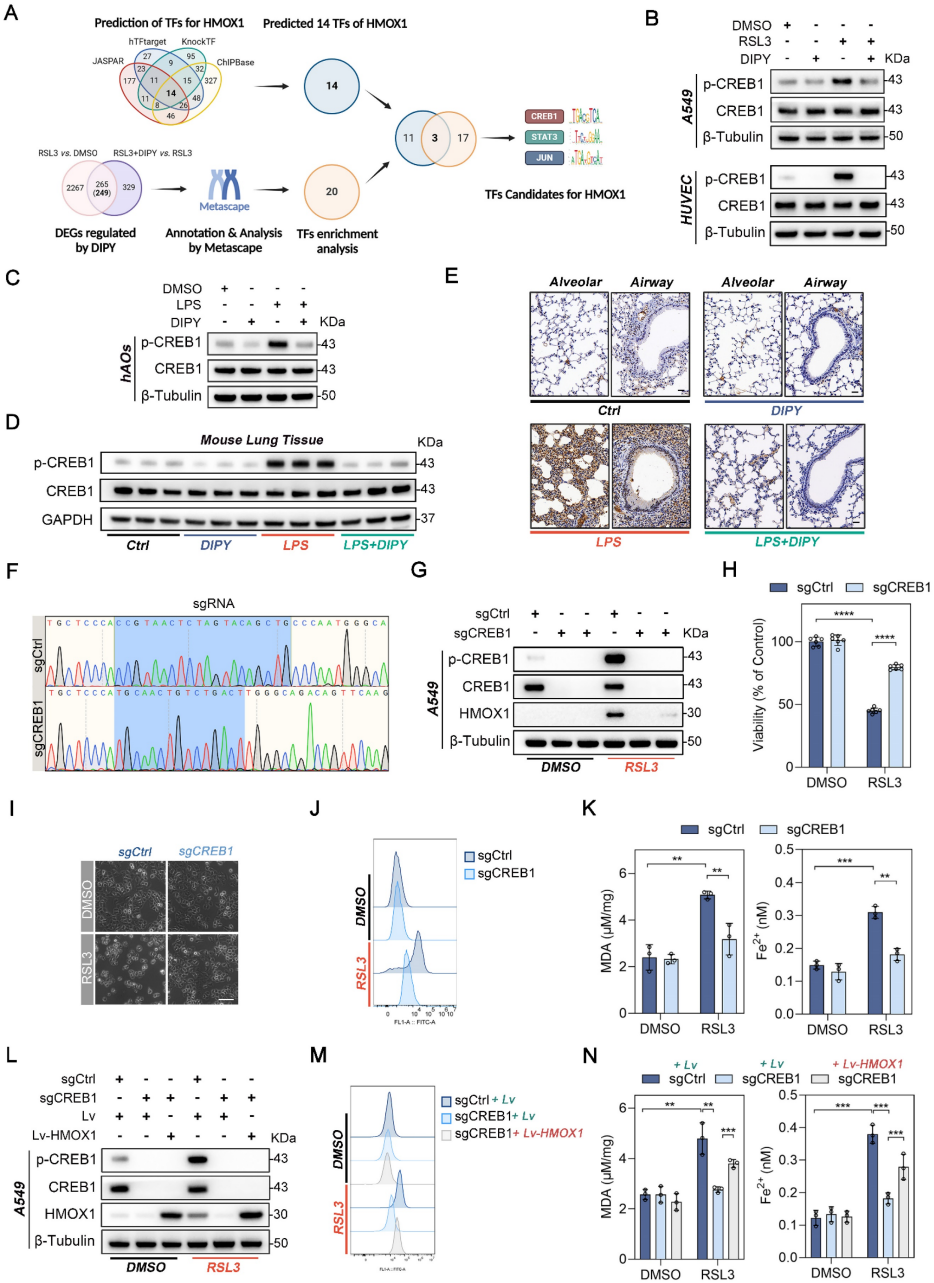
** DIPY suppresses HMOX1 through the deactivation of CREB1.** (**A**) Schematic illustration for screening TFs for the human *HMOX1* gene. (**B**) A549 and HUVEC cells were treated with RSL3, DIPY, and RSL3 with DIPY for 8 hours as shown in Figure 1. Cell lysates were collected and analyzed with the indicated antibodies (n = 3 per group). (**C**) hAOs were treated with LPS, DIPY, and LPS with DIPY as shown in Figure S3 (n = 5 per group). The lysates were subjected to immunoblotting with the indicated antibodies. (**D-E**) Western blot (**D**) and IHC assays (**E**) examined the expression of p-CREB1 in LPS-induced ALI mouse models with or without DIPY treatment as shown in Figure 2 (n = 6 per group). Scale bar, 50 μm. (**F-K**) CREB1 knockout A549 cell lines (sgCREB1) and the control cells (sgCtrl) were treated with RSL3 (5 μM) for 8 hours (n = 3 per group). Confirmation of CREB1 knockout via sequencing (**F**). Cell lysates were subjected to immunoblotting with the indicated antibodies (**G**). Cell viability assessment (**H**). Representative phase-contrast images of cells (**I**). Scale bar, 100 μm. L-ROS levels measurement (**J**). MDA contents and Fe^2+^ levels (**K**). (**L-N**) sgCREB1 A549 cells or the control cells were infected with lentiviruses carrying Lv-HMOX1 or Lv. The cells were treated with RSL3 (5 μM) for 8 hours (n = 3 per group). Cell lysates were subjected to immunoblotting with the indicated antibodies (**L**). L-ROS levels (**M**). MDA contents and Fe^2+^ levels (**N**). Results are shown as mean ± SD of 3-6 independent experiments. Statistical significance is indicated as **P < 0.01; ***P < 0.001; ****P < 0.0001.

To elucidate the mechanism by which DIPY suppresses HMOX1, we first performed the *in silico* analysis of the *HMOX1* gene using four public databases (JASPAR, hTFtarget, KnockTF, and ChIPBase database). Fourteen candidate transcriptional factors (TFs) for the *HMOX1* gene were identified (Figure 6A). Furthermore, DIPY-regulated 249 DEGs in our RNA-Seq data were annotated and analyzed by Metascape, and 20 TFs were enriched (Figure 6A) 19. Subsequently, a Venn diagram analysis on these two TF datasets was performed, and 3 TFs (CREB1, STAT3, and JUN) have been screened (Figure 6A and Table S1). Among these TFs, only CREB1 and JUN can be activated by RSL3 and reversed by DIPY treatment (Figure 6B and Figure S7A). However, knockdown CREB1, but not JUN, led to a significantly downregulated expression of HMOX1 (Figure S6B). Moreover, DIPY markedly reduced the phosphorylation of CREB1 in LPS-challenged hAOs, LPS-induced ALI, and CLP-induced sepsis mice models (Figure 6C-E and Figure S7C-D). Therefore, it is likely that DIPY inhibits HMOX1 through suppression of CREB1.

To further examine whether CREB1 mediated the expression of HMOX1 during ferroptosis, we treated A549, HUVEC, and BEAS-2B cells with 666-15, a potent and selective inhibitor of CREB1. As expected, inhibition of CREB1 dramatically reduced the expression of HMOX1 induced by RSL3 (Figure S7E). Notably, 666-15 effectively prevented RSL3-induced cell death, lipid peroxidation (L-ROS and MDA), and iron accumulation (Figure S7E-H). Moreover, the CRISPR/Cas9 system was performed to knock out CREB1 in the A549 cells (Figure 6F-G). Similar to the pharmacologic inhibition of CREB1, the genetic deletion of CREB1 led to a noticeable inhibition of HMOX1 expression and ferroptosis induced by RSL3 (Figure 6G-K). In addition, we re-expressed HMOX1 in sgCREB1 A549 cells by transfecting the *HMOX1* gene. The forced expression of HMOX1 attenuated ferroptosis resistance in sgCREB1 cells, which was related to increased cell death, oxidative damage (L-ROS and MDA), and Fe^2+^ accumulation (Figure 6L-N and Figure S7I-J). Together, these results suggested that DIPY inhibits ferroptosis through the CREB1/HMOX1 pathway in lung epithelial and endothelial cells.

Inhibition of CREB1 by both pharmacologic and genetic strategies led to the decrease of HMOX1 mRNA in response to RSL3, suggesting that CREB1 regulates the expression of HMOX1 at the transcriptional level (Figure S8A-B). We then analyzed the promoter region (-2000 to +200) of human *HMOX1* using JASPAR, which predicted a possible binding region of CREB1 (-685/-672, TGTGACACCACTG; with score > 10.0; Figure S8C). Next, we cloned the human *HMOX1* gene promoter (-847 to +65) into the pGL3 luciferase reporter vector and evaluated its promoter activity. Interestingly, although the activity of the wild-type HMOX1 promoter construct was enhanced by RSL3 treatment, the mutation of the potential CREB1 binding site did not change its activity (Figure S8D). This result suggested that the potential binding site in the promoter of the *HMOX1* gene is not responsible for the upregulation of HMOX1 induced by RSL3.

Next, by analyzing two Chromatin immunoprecipitation (ChIP)-Seq databases (ChIP-Atlas and ENCODE), we found two regions upstream of the promoter (around -9843/-7346 and -4920/-3111) with enrichment of CREB1-binding peaks, which were also highly enriched the active enhancer marker H3K27Ac (Figure S8E). Three putative CREB1 binding sites (#1, -3570/-3563; #2, -4519/-4512; #3, -7488/-7481) were found in these two regions by using the JASPAR database (Figure S8F). We confirmed that pCREB1 binds to all these three sites using ChIP-PCR assays (Figure S8G). Subsequently, the ChIP-qPCR assay revealed dramatically increased occupancy of CREB1 on these regions in RSL3-induced cells, compared to the control cells (Figure S8H). Moreover, CREB1 deletion abolished the binds of CREB1 and these enhancers even under RSL3-treatment conditions (Figure S8I). Collectively, these data suggested that DIPY inhibits ferroptosis by suppressing the CREB1/HMOX1 pathway.

### DIPY inhibits the CREB1/HMOX1 pathway through directly binding to and activating SOD1

**Figure 7:**
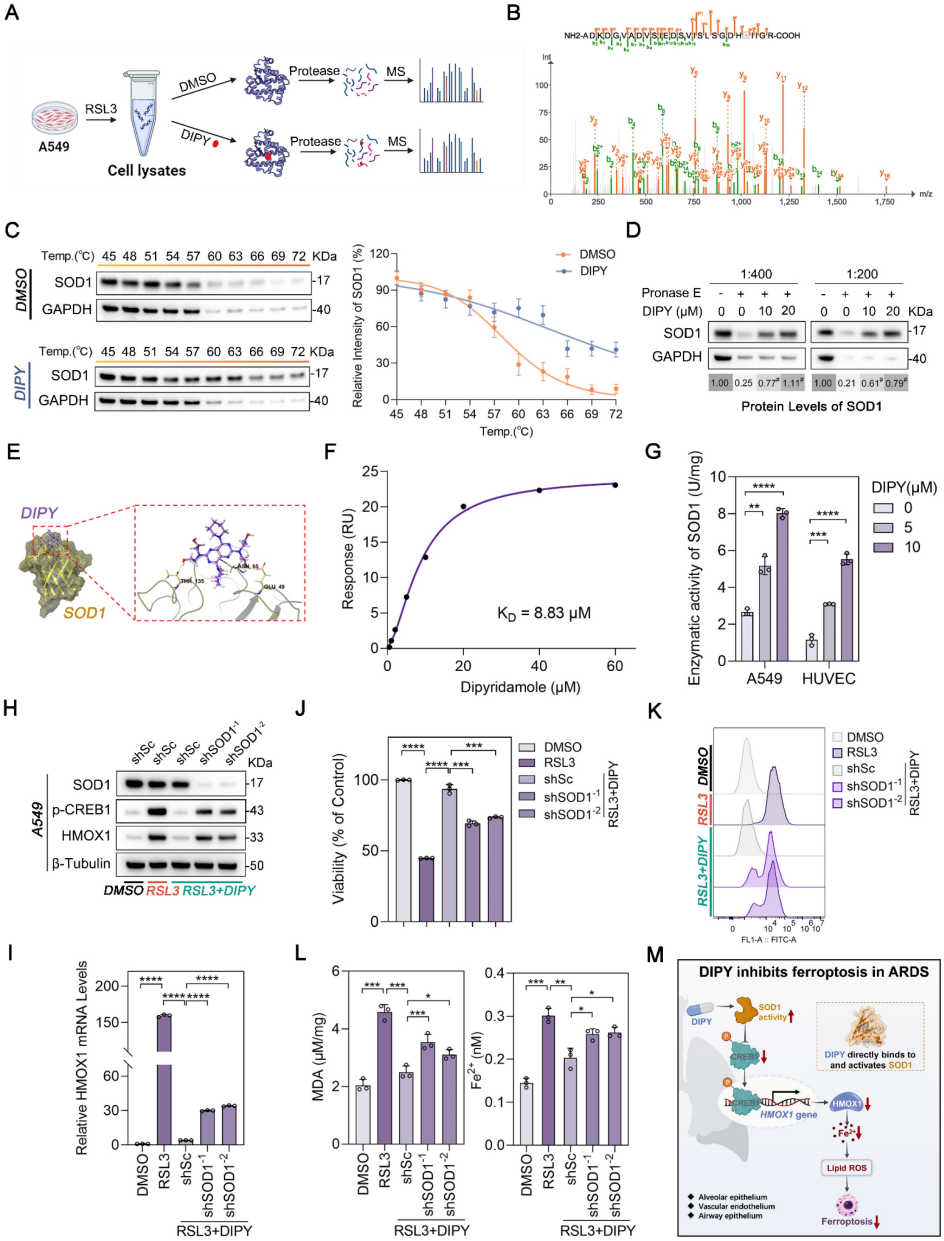
** DIPY inhibits the CREB1/HMOX1 pathway through directly binding to and activating SOD1.** (**A**) Schematic diagram illustrating the LiP-MS assay used to identify the direct target of DIPY. A549 cells were pretreated with RSL3 (5 μM) for 8 hours. The lysates were collected and incubated with DIPY, followed by digestion with proteinase K for 10 minutes, and then analyzed using MS. (**B**) MS analysis showing the fragmentation pattern of SOD1 peptide. The spectrum includes the identification of b-ions (green) and y-ions (orange) corresponding to the amino acid sequence of the SOD1 protein. (**C** and **D**) A549 cells were treated with RSL3 (5 μM) for 8 hours. The harvested cell lysates were incubated with DIPY (10 μM) or DMSO for 30 minutes, followed by CETSA (**C**) and DARTS (**D**) analysis. (**E**) Three-dimensional binding mode diagrams between DIPY and SOD1. (**F**) SPR analysis of DIPY-binding to SOD1. K_D_ = 8.83 μM. (**G**) A549 and HUVEC cells were treated with RSL3 (5 μM) and DIPY (0, 5 μM, 10 μM). The enzymatic activity of SOD1 was examined. (**H-L**) A549 cells were infected with lentivirus expressing shRNAs target SOD1 (shSOD1^-1^ and shSOD1^-2^) or a scrambled sequence (shSc). The cells were incubated with RSL3 (5 μM), with or without DIPY (10 μM), for 8 hours. Cell lysates were subjected to immunoblotting with the indicated antibodies (**H**). The level of HMOX1 mRNA was analyzed using qRT-PCR (**I**). Cell viability assessment (**J**). L-ROS levels measurement (**K**). MDA contents and Fe^2+^ levels (**L**). (**M**) Schematic illustration of how DIPY suppresses ferroptosis in pulmonary epithelial and endothelial cells by regulating the SOD1/CREB1/HMOX1 pathway in ARDS. Results are shown as mean ± SD of at least 3 independent experiments. Statistical significance is indicated as *P < 0.05; **P < 0.01; ***P < 0.001; ****P < 0.0001.

A limited proteolysis-mass spectrometry (LiP-MS) assay was employed to identify proteins that could directly bind to DIPY (Figure 7A). As shown in Figure S9A-B, 58 peptides belonging to 47 proteins were identified (Table S2). Reactome pathway analysis of the different peptides revealed that the “Cellular responses to stress” pathway was enriched (Figure S9C). Among 11 proteins of this enriched pathway, SOD1, a well-known antioxidant enzyme, aroused our great interest (Figure 7B and Table S3).

To demonstrate the interaction of DIPY and SOD1, we initially conducted a cellular thermal shift assay (CETSA) to assess the thermal stability of SOD1 in A549 cells upon DIPY treatment. As shown in Figure 7C, DIPY elevated the thermal stability of SOD1 compared with the control group.

Furthermore, drug affinity responsive target stability (DARTS) experiments indicated that DIPY stabilized SOD1 by increasing resistance to proteolysis (Figure S9D and Figure 7D). Also, molecular docking demonstrated that DIPY may bind to SOD1 through several conserved hydrogen bonds (Figure 7E). Moreover, SPR analysis indicated that DIPY is directly bound to SOD1 (K_D_ = 8.83 μM; Figure 7F). Notably, DIPY significantly elevated the activity of SOD1 in A549 and HUVEC cells and the lung tissues of ARDS mice (Figure 7G and Figure S9E-F). Therefore, these results demonstrated that DIPY directly binds to and activates SOD1 in lung epithelial and endothelial cells.

To evaluate whether SOD1 mediates the anti-ferroptoic effect of DIPY, we knocked down SOD1 using two shRNAs in A549 cells. As shown in Figure 7H-L, the knockdown of SOD1 resulted in a substantial elevation in CREB1 phosphorylation, HMOX1 expression, and ferroptosis sensitivity. Besides, SOD1 was ectopically overexpressed in HUVEC cells and verified by western blot and qRT-PCR (Figure S9G-H). As expected, SOD1 overexpression attenuated the increased CREB1 phosphorylation, HMOX1 expression, and ferroptosis induced by RSL3 (Figure S9G-L). Collectively, these data suggested that DIPY directly binds to and activates SOD1, which then induces ferroptosis resistance by inhibiting the CREB1/HMOX1 pathway in lung epithelial and endothelial cells (Figure 7M).

### Clinical evaluation of DIPY in patients with ARDS indicates beneficial effects

**Figure 8:**
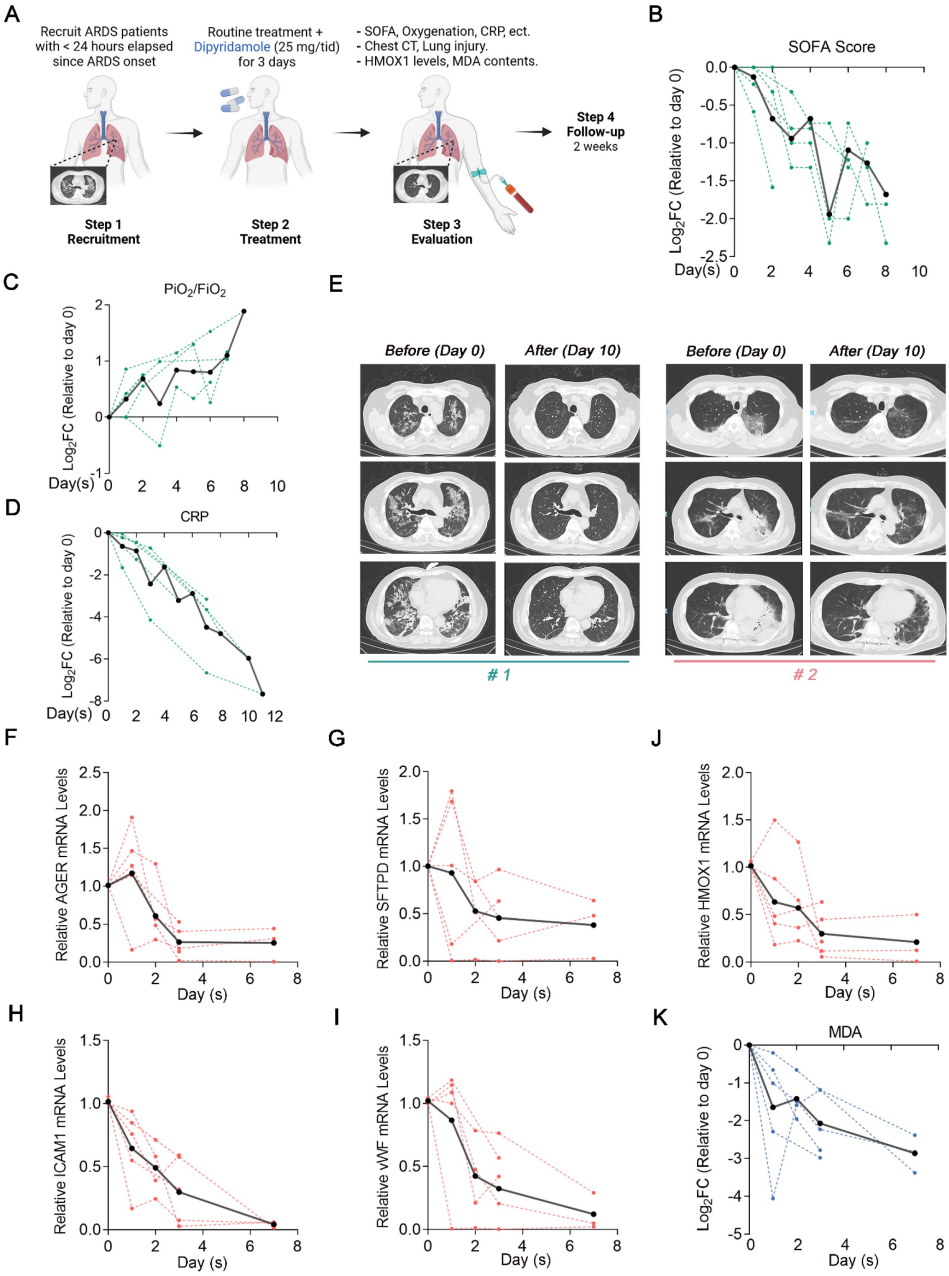
** Clinical evaluation of DIPY in patients with ARDS indicates beneficial effects.** (**A**) Clinical study flow chart. Peripheral blood sampling was performed at baseline before the first dose of DIPY and 1, 2, 3, and 7 days after the initiation of DIPY adjunctive treatment, respectively. Patients were monitored for 14 days after completion of DIPY treatment. (**B-D**) Clinical parameters post-DIPY treatment. SOFA score at indicated time points after DIPY treatment, normalized to day 0 (pre-DIPY treatment, **B**). PiO_2_/FiO_2_ ratio at indicated time points after DIPY treatment, normalized to day 0 (**C**). CRP levels at indicated time points after DIPY treatment, normalized to day 0 (**D**). (**E**) Chest computed tomography scans of two subjects (#1 and #2) before DIPY treatment (Day 0) and 10 days after DIPY treatment (Day 10). (**F-J**) The mRNA levels of indicated injury markers (**F** for AGER,** G** for SFTPD,** H** for ICAM1, and **I** for vWF) and HMOX1 (**J**) in plasma from ARDS patients at indicated time points before and after DIPY treatment were analyzed using qRT-PCR. (**K**) MDA contents at indicated time points after DIPY treatment, normalized to day 0. (**B-D, F-K**) Data is shown with individual patient responses as dotted lines and the mean of all subjects as solid lines.

Based on these preclinical data, we designed and launched a proof-of-concept trial (ChiCTR2300078059) to test whether this newfound insight could be harnessed for treating human ARDS. We recruited 5 patients with moderate-to-severe ARDS which < 24 h elapsed since ARDS onset. The baseline characteristics of the patients are described in Table S4. Patients received 25 mg of DIPY thrice daily for 3 consecutive days, administered concomitantly with routine treatment. Baseline blood samples and the blood samples of 1, 2, 3, and 7 days after DIPY adjunctive therapy were obtained. Observed clinical effects and laboratory parameters include improved respiratory symptoms, radiographic evaluations, and resolution of lung injury (Figure 8A).

The patients have experienced an improvement in respiratory symptoms after treatment without significant antiplatelet, anticoagulant, and antihypertensive effects (Figure S10A-C). Marked reductions in Sequential Organ Failure Assessment (SOFA) score, increased oxygenation assessed by PaO_2_/FiO_2_ ratio, and decreased inflammatory (C-reactive protein, CRP) were seen in almost all clinical subjects (Figure 8B-D). Chest computed tomography scans indicated that patients showed absorption of pulmonary pathological changes after DIPY adjunctive therapy (Figure 8E and Figure S10D). Also, DIPY adjunctive treatment significantly mitigated the injury of lung epithelial cells (indicated by AGER and SFTPD) and vascular endothelial cells (indicated by ICAM1 and vWF; Figure 8F-I). Notably, we observed a consistent decrease in HMOX1 mRNA expression over time in the majority of peripheral blood samples following DIPY treatment, aligning with the findings observed in ARDS mice models (Figure 8J). As expected, the serum levels of MDA (a specific *in vivo* marker for ferroptosis 20) were significantly downregulated by DIPY treatment in the patients (Figure 8K). As of the most recent data cut (July 2024), all five patients were clinical resolution, and the study remained ongoing. Therefore, our data provided proof-of-concept evidence establishing inhibition of ferroptosis by DIPY as a viable clinical strategy for combating ARDS.

## Methods

Sex as a biological variable. Male and female human ARDS samples were analyzed. Male mice were used in all mouse studies. In this study, sex was not considered as a biological variable.

### Patient selection and clinical specimens

The clinical study, according to the Declaration of Helsinki principles, was approved by the Clinical Medical Research Ethics Committee of the First Affiliated Hospital of Anhui Medical University (No. PJ-2023-09-07) and registered in the Chinese Clinical Trial Registry (ChiCTR2300078059). A total of 5 patients diagnosed with ARDS according to the 2012 Berlin ARDS diagnostic criteria, or the New Global Definition of ARDS, were recruited for the proof-of-concept clinical trial, with informed consent obtained from each participant. Blood samples were collected before and after DIPY treatment, and the expression of HMOX1, the MDA levels, and the injury markers of lung epithelium and endothelium were measured. Baseline information for all enrolled participants is provided in **Table S4**.

### Reagents and antibodies

FDA-approved drug library (#Z705684), DIPY (#S1895), STS (#S1421), Rapa (#S1039), and 666-15 (#S8846) were obtained from Selleck Chemicals. RSL3 (#HY-100218A), Erastin (#HY-15763), Ferrostatin-1 (#HY-100579), and ZnPP (#HY-101193) were purchased from Med Chem Express. LPS (#2630) and DMSO (#D2650) were obtained from Sigma-Aldrich. The antibodies used in this study are detailed in **Table S5**.

### Cell lines and cell culture

A549 (Shanghai Institute of Life Sciences), BEAS-2B (American Type Culture Collection, ATCC), and HEK293T (ATCC) cells were cultured in a Dulbecco's modified Eagle's medium (DMEM, Hyclone). HUVEC (Bioogenetech) cells were cultured in Roswell Park Memorial Institute-1640 complete medium (RPMI-1640, Hyclone). All culture mediums were supplemented with 10% fetal bovine serum (FBS, Life Technologies), 100 units/mL penicillin, and 100 μg/mL streptomycin, and incubated in a 5% CO_2_ atmosphere at 37 °C.

### Cell viability

Cell viability was assessed using the Cell Counting Kit-8 (CCK-8, #C0005, TargetMol) following the manufacturer's instructions. Cells were seeded in 96-well microplates and incubated overnight. After the treatments, the cells were incubated with CCK-8 for 1 h. Absorbance at 450 nm was then measured using a microplate reader (ELX808, BioTek) to evaluate cell viability.

### Drug screening

The drug compound library (#Z705684, Selleck), consisting of 259 FDA-approved compounds for drug screening, was obtained from Selleck Chemicals. The final concentration of compounds used in this experiment was 10 μM. A549 cells were seeded into 96-well plates at a density of 5,000 cells per well and incubated overnight. The cells were treated with the compound library (10 μM) with or without RSL3 (5 μM) for 8 h. Following treatment, cell viability was measured using the CCK-8 reagent. Ferrostatin-1 was used as a positive control to identify compounds that inhibit ferroptosis.

### Establishment and culture of hAOs

To generate hAOs, adjacent normal mucosal tissues were obtained with informed consent from patients diagnosed with NSCLC undergoing routine surgeries at the First Affiliated Hospital of Anhui Medical University. The study protocol received approval from the Ethical and Scientific Committee of the First Affiliated Hospital of Anhui Medical University (No. 2023669). The clinical information for the donors is summarized in **Table S6**. The hAOs were cultured following Sachs' method 21. Briefly, solid lung tissues obtained from NSCLC patients were washed with AdDF medium (Advanced DMEM/F12 containing 10 mM HEPES, 1× GlutaMAX, and 1× penicillin-streptomycin), minced into 1-2 mm³ fragments, and digested with 2 mg/mL collagenase in AdDF medium at 37 °C for 30 min. The suspension was filtered through a 100 µm filter and centrifuged at 200×g for 5 min. Cell clusters were lysed in 1 mL red blood cell lysis buffer for 5 min, stopped with AO medium (as detailed in **Table S7**), and centrifuged again at 200×g for 5 min. The lung cell clusters were suspended in the lung organoid medium and solidified on Matrigel (Corning). After gelation, the Matrigel-cell mixture was incubated with a lung organoid medium at 37 °C. Medium was replaced every 3 days, and organoids were passaged biweekly.

Organoid viability was assessed using Acridine Orange and Propidium Iodide Staining (AO/PI, Santa Cruz). Organoids were digested, and the suspension was collected. A 10 µL aliquot of the suspension was mixed with 10 µL of AO/PI solution and incubated for 20 min at 4 °C. The mixture was then placed on a glass slide. Fluorescence was visualized using a cell fluorescence analyzer (Alit Biotech). Organoid viability was determined by the ratio of green to red fluorescence.

### RNA interference, lentivirus infection and CRISPR-Cas9

All small interfering RNA (siRNA) oligonucleotides were synthesized by GenePharma (Shanghai, China). Cells were transfected by Lipofectamine RNAiMax (Thermo Fisher) according to the product instructions.

All lentivirus vectors were obtained from GenePharma, including the LV6 lentiviral plasmid expressing HMOX1 cDNA, SOD1 cDNA, and the corresponding empty plasmid, as well as the LV-2N shRNA expression vector targeting HMOX1, SOD1, and the control scrambled shRNẠ (shSc). Stable cell lines were selected using puromycin (Solarbio) at the appropriate concentration. Lentivirus infection efficiency was validated by western blot and qRT-PCR.

The single guide RNA (sgRNA) targeting CREB1 was designed using the OpenAccess software program CRISPR and synthesized by Tsingke Biotech Co., Ltd. The oligos were selected and cloned into pLentiCRISPR v2 plasmid (Addgene). Recombinant plasmids, along with psPAX2 and pVSVG, were co-transfected into HEK293T cells. Following lentivirus transfection, cells were selected with puromycin for one week and then transferred into 96-well plates to obtain individual clones. Positive clones were further characterized to confirm CREB1 knockout by western blot and sequencing.

The sequences of the siRNA, shRNA, and sgRNA are listed in **Table S8.**

### Western blot analysis and qRT-PCR

Western blot analysis and qRT-PCR were performed as previously described 22. The blots were detected by chemiluminescence and analyzed using the Image J software (https://imagej.nih.gov/ij/). All primer sequences used in qRT-PCR were provided in **Table S9.**

### IF assay

The IF assays were performed as previously described 23. Briefly, the samples of cells and hAOs were fixed, permeabilized, blocked, and incubated with primary and fluorescently labeled secondary antibodies. Nuclei were stained with DAPI (Beyotime Biotechnology). Images were captured using a Zeiss LSM980 + Airyscan Laser Confocal Microscope.

### RNA-Seq analysis and data analysis

RNA-seq was conducted by Aksomics (Shanghai, China). Total RNA was isolated from A549 cells using the TRIzol^®^ Reagent Kit (Thermo Fisher Scientific). The RNA was then identified and quantified using a NanoDrop instrument, and 1-2 μg of total RNA was used for RNA-seq library construction. mRNA was enriched using NEB Next^®^ Poly(A) mRNA Magnetic Isolation Module (New England Biolabs) and RNA-seq libraries were prepared using the KAPA Stranded RNA-Seq Library Prep kit (Illumina) according to the manufacturer's protocol. The RNA-seq libraries were assessed using an Agilent 2100 Bioanalyzer, and all samples were sequenced on an Illumina NovaSeq 6000. Genes with a |Fold change| ≥ 1.5 and a p-value ≤ 0.05 were identified as DEGs. GSEA was performed using the GSEA tool (https://www.gsea-msigdb.org/gsea/index.jsp). Transcription factor enrichment analyses were performed using the online tool Metascape (www.metascape.org) with default parameters 19. The RNA-Seq data generated in this study are publicly available in the NCBI Gene Expression Omnibus (GEO) database (GEO GSE272163).

### Reporter constructs and dual-luciferase reporter assay

A DNA fragment (-847 to +65) containing the promoter region of the human *HMOX1* gene was inserted into the pGL3 Basic Vector (Promega, Madison, WI). The potential CREB1 binding site within the HMOX1 promoter was mutated using the Q5^®^ Site-Directed Mutagenesis Kit (NEB, Ipswich, MA, USA). The primers used for constructing the recombinant plasmids are listed in **Table S10**. To investigate the effect of CREB1 activation on HMOX1 promoter activity, HEK293T cells were co-transfected with either the wild-type or mutated promoter constructs, along with pRL-TK (20 ng, Promega) as an internal reference. Luciferase activity was measured using the Dual-Luciferase Reporter Assay System (Promega) according to the manufacturer's instructions.

### ChIP assay

ChIP assays were conducted using the SimpleChIP® Plus Enzymatic Chromatin IP kit (Cell Signaling Technology), following the manufacturer's protocol. After the indicated treatment, cells were cross-linked with 1 % formaldehyde for 10 min, lysed with sodium dodecyl sulfate lysis buffer, and ultrasonicated for 50 min. The lysates were incubated overnight at 4 ℃ with the p-CREB1 (Ser133) antibody. The immunoprecipitated DNA was then purified and analyzed using PCR or qRT-PCR with specific primers. The primer sequences are listed in **Table S11**.

### Lipid-ROS assay, MDA assay, and Iron assay

Lipid-ROS assay and MDA assay were performed as previously described 24. The Fe^2+^ levels were measured using an iron assay kit (Abcam, ab83366) according to the manufacturer's protocol. The quantitative analysis of the lipid-ROS assay is presented in Figure S11.

### TEM analysis

Cells and airway organoids were cultured and treated with the indicated drugs for a corresponding time. After washing with cold PBS, the samples were fixed with 4% paraformaldehyde, followed by 2.5% glutaraldehyde at 4 °C. Lung tissues were removed from mice and immediately fixed in 2.5% glutaraldehyde. Samples were then dehydrated, embedded, sectioned, and stained with uranyl acetate and lead citrate. TEM images were captured using a transmission electron microscope (JEM1400).

### LiP-MS assay

LiP-MS assay was performed by SpecAlly Life Technology Co., Ltd (Wuhan, China) following Liu's method 25. In brief, cell pellets were homogenized in lysis buffer and centrifugated at 12000 rpm for 5 min at 4 ℃. Protein concentrations of samples were measured using a BCA assay kit (Beyotime Biotechnology). Subsequently, 500 μg of total protein was incubated with 10 μM DIPY or DMSO for 10 min at 25 ℃. Proteinase K (5 μg, Sigma-Aldrich) was added to each sample and incubated for an additional 10 min. The reaction was then stopped by heating at 98 ℃ for 3 min. Samples were subjected to limited proteolysis to generate structure-specific protein fragments. Digestions were completed by treating with trypsin (Promega) at an enzyme-substrate ratio of 1:50 at 37 ℃ overnight, and the reaction was stopped by adding trifluoroacetic acid. Sample supernatant was collected and immediately analyzed using an Orbitrap Q Exactive Plus mass spectrometer. The obtained raw data was processed through a rigorous computational workflow, including Data Acquisition, Peptide Label-Free Quantitation, and screening for DIPY-binding proteins. Finally, 58 peptides corresponding to 47 proteins were obtained for further validation, and the Reactome pathway analysis was performed. The data is presented in **Tables S2 and S3.**

### CETSA assay

A549 cells were pretreated with RSL3 (5 μM) for 8 h. After collection, the cells were divided into equal volumes. The aliquoted cell suspensions were incubated with DIPY (10 μM) or DMSO for 30 min and then subjected to thermal digestion at a range of temperatures (45, 48, 51, 54, 57, 60, 63, 66, 69, and 72 °C) for 3 min. The samples were centrifugated at 20,000×g for 10 min at 4 °C, and the supernatants were collected for western blot analysis.

### DARTS assay

A549 cells were lysed and collected following RSL3 treatment (5 μM, 8 h). The supernatant was incubated with varying concentrations of DIPY or DMSO for 30 min at room temperature. Then, indicated concentrations of Pronase E (#HY-114158A, Med Chem Express) were supplemented into the aliquoted cell lysates for 30 min. Proteolysis was halted by boiling the samples after adding protein loading buffer for subsequent SDS-PAGE or western blot analysis.

### Molecular docking

The crystal structures of target proteins were retrieved from the RCSB Protein Data Bank (https://www.rcsb.org; SOD1: 8GSQ). Molecular docking between DIPY and the SOD1 protein was performed using Schrödinger software as follows:

The three-dimensional (3D) structure of SOD1 underwent protein preprocessing, including regeneration of native ligand states, H-bond assignment optimization, protein energy minimization, and water molecule removal. The 3D structure of DIPY was processed from its two-dimensional (2D) structure using the LigPrep module. The Receptor Grid Generation module defined the active site of SOD1 by setting an enclosing box around the predicted binding site. Molecular docking was then performed in the Ligand Docking module with extra precision (XP), and the corresponding GScore was recorded. Additionally, the active sites of DIPY and SOD1 were analyzed using MM-GBSA calculations.

### SPR analysis

The binding affinities of DIPY for the SOD1 protein were evaluated using an SPR-based Biacore T200 apparatus (GE Healthcare). A CM5 sensor chip was used to immobilize 15,000 RU of the human SOD1 protein (#HY-P71048, MCE) onto the sensor surface via standard amine coupling at 25 °C in the PBS running buffer. Gradient concentrations of DIPY were injected into the fluid flow system at a flow rate of 30 μL/min to evaluate the binding affinity. The equilibrium dissociation constant (K_D_) of the DIPY-SOD1 complexes was calculated using the Biacore T200 apparatus Evaluation Software.

### The enzymatic activity of SOD1

SOD1 enzymatic viability was assessed using a Cu/Zn-SOD assay kit with WST-8 (#S0103, Beyotime). In brief, the supernatant of cell or tissue samples was collected after homogenization at 4 °C and incubated with WST-8/enzyme working solution for 30 min at 37 °C and measured at 450 nm using a microplate reader.

### ARDS mouse models and therapeutic interventions

All animal experimental procedures were approved by the Experimental Animal Ethical Committee of Anhui Medical University (No. LLSC-20221112). Male adult C57BL/6J mice (8-10 weeks) used in this study were purchased from Gempharmatech Co., Ltd (Nanjing, China) and maintained under specific-pathogens-free conditions. In the model of LPS-induced ALI, mice were challenged with LPS via intratracheal instillation at either a low dose (2.5 mg/kg) or a lethal dose (20 mg/kg). Lethal-dose LPS was only used for testing survival. To ensure even distribution of LPS in the lungs, the mice were gently shaken from side to side. The prophylactic effect of DIPY was evaluated by administering an intraperitoneal injection of 10 mg/kg 1 h before LPS installation. After 24 h of the LPS exposure, the mice were euthanized, and lung tissue and BALF were collected for further analysis.

For another ARDS mice model (CLP-induced sepsis), male adult C57BL/6J mice (8-10 weeks) were subjected to CLP surgery to induce sepsis as described by Park 26. Mice received an oral administration of DIPY (10 mg/kg) or Saline 3 days before CLP surgery. For the CLP procedure, mice had their abdominal hair removed and were anesthetized with isoflurane. The peritoneal cavity was then opened, and the cecum was exteriorized and ligated at different points distal to the ileocecal valve using a nonabsorbable 3-0 suture. To induce mid-grade sepsis (mild CLP), approximately 50% of the cecum was ligated. To induce lethal sepsis (lethal), approximately 75% of the cecum was ligated (lethal sepsis was only used for testing survival). The distal end of the cecum was then perforated using a 20 G needle, and a small drop of feces was extruded. The cecum was then relocated back into the peritoneal cavity, and all animals received subcutaneous fluid resuscitation with 1 mL of sterile saline. Eighteen hours after the CLP surgery, the mice were sacrificed for molecular and histological analysis.

For subsequent studies, the C57/BL6 mice were administered HMOX1-overexpressed lentivirus (Lv-HMOX1) or the empty vector (Lv) via intratracheal instillation for seven consecutive days. On day 8, the indicated mice were challenged with LPS (2.5 mg/kg) with or without DIPY (10 mg/kg) pretreatment (n = 6 per group). After 24 h of the LPS challenge, the mice were sacrificed, and the BALF and lung tissue were collected for further analysis.

### H&E and immunohistochemical (IHC) staining

H&E and IHC staining were performed as previously described 23. The hAOs and lung tissue were fixed in 4% PFA, paraffin-embedded, and sectioned into 4-μm-thick histology slices for histological analysis. H&E staining of paraffin sections was performed as standard procedures. The degree of lung inflammation and damage was evaluated 27. For IHC staining, lung tissue sections were dewaxed, rehydrated, and subjected to antigen retrieval. Endogenous peroxidase activity was blocked with 3% H_2_O_2_. After blocking with 5% bovine serum albumin, the sections were incubated with primary antibodies overnight at 4 °C. The following day, the sections were washed with PBS, incubated with secondary antibodies at room temperature for 30 min, and developed using Diaminobenzidine (DAB, #B011, Ebiogo).

### Enzyme-linked immunosorbent assay (ELISA) assay

The expression levels of TNF-α, IL-1β, and IL-6 in BALF from mouse models of ARDS were detected by ELISA kits (R&D Systems) according to the corresponding kit instructions.

### Lung W/D weight ratio

The W/D ratio of lung tissues was used to assess the degree of pulmonary edema. The left lower lobe of the mouse was excised and immediately weighed as wet weight (W), and then placed in a constant temperature oven at 60 ℃ for 48 h for dry weight (D). Ultimately, the W/D ratios could be calculated.

### The CT evaluation

The LPS-induced ARDS mice were anesthetized, and the lungs were scanned at 80 kV and 150 mA using a 64-multislice spiral CT scanner (GE Brightspeed™ RT 16 Elite CT scanner). Image acquisition was synchronized to the respiratory cycle and reconstructed with a slice thickness of 0.625 mm.

### Bacterial burden

Blood and lung tissues of mice were aseptically harvested 18 h after CLP surgery. Lung tissues were homogenized in sterile PBS, and the homogenates were diluted 10-fold with sterile PBS. Blood samples and lung homogenates were immediately plated on blood agar plates and cultured in an aerobic environment at 37 ℃ for 24 h. The following day, colony-forming units (CFUs) were counted to assess the bacterial burden across different samples.

### Statistics

All statistical analyses were performed using GraphPad Prism software (version 6.0) and IBM SPSS software (version 23.0, IBM Corp). Data are presented as the mean ± SD. Statistical significance was determined using a 2-tailed Student's *t*-test or 1- or 2-way ANOVA followed by multiple post hoc tests. All experiments were independently performed at least three times. A *P* value of less than 0.05 was considered statistically significant.

## Discussion

Emerging evidence indicates that inhibiting ferroptosis represents an effective strategy for mitigating pulmonary inflammation and tissue damage in preclinical models of ARDS 6, 28. However, currently, there are no FDA-approved ferroptosis-related drugs for clinical applications in treating ARDS. In this study, we identified DIPY as a powerful inhibitor of ferroptosis in lung epithelial and endothelial cells through a screen of 259 FDA-approved drugs. The anti-ferroptotic and therapeutic effects of DIPY were further verified in two ARDS mice models and hAOs. Notably, a proof-of-concept trial demonstrated beneficial effects in patients with ARDS following DIPY adjunctive therapy. Mechanistically, we illustrated that DIPY directly binds to and activates SOD1, which in turn suppresses ferroptosis by inhibiting the CREB1/HMOX1 pathway.

In recent years, drug repositioning strategies have received widespread attention due to their inherent advantages, including accelerated drug development times, and prior knowledge about the safety, dosage, and toxicity profiles 29-32. For example, statins, traditionally used to reduce cholesterol and improve cardiovascular diseases, have been developed as an anti-cancer agent and are currently under interventional clinical trials 33-35. DIPY, an inhibitor of phosphodiesterases, is routinely used to inhibit platelet aggregation and manage platelet-mediated thrombotic diseases 36-38. In the present study, based on studying cell lines, mice models, hAOs, and clinical trials, we found that DIPY could suppress ferroptosis and then benefit patients with ARDS, further implying the significance of drug repurposing in developing ARDS therapies. Viral and bacterial pneumonia is one of the most common risk factors for ALI/ARDS 1. Most critically ill patients infected with severe acute respiratory syndrome coronavirus 2 (SARS-CoV-2) develop an extensive inflammatory insult that is driven by COVID-19 pneumonitis leading to ARDS 39, 40. Interestingly, it has been shown that DIPY has therapeutic effects in severely ill COVID-19 patients by suppressing SARS-CoV-2 replication and reducing D-dimer levels 41. Considering that COVID-19 induces ferroptosis and oxidative stress in human endothelial cells 42, our findings may provide additional insight into the beneficial effects of DIPY in treating COVID-19 patients. We proposed that DIPY may offer pulmonary protection by inhibiting ferroptosis in the lung epithelium and endothelium during SARS-CoV-2 infection. It should be noted that DIPY may not be suitable for preventing ARDS caused by circulatory shock 43, as DIPY can potentially lead to a decrease in blood pressure 44. In addition to pulmonary diseases, ferroptosis has been implicated in the development of other conditions, such as ischaemic organ injuries and neurodegeneration 45-48. Therefore, DIPY may have potential applications in treating these ferroptosis-related diseases, warranting further investigation.

Using RNA-Seq and subsequent systematic analysis, we illustrated that DIPY suppresses ferroptosis due to HMOX1 inhibition. It's well-known that HMOX1, which catalyzes the cleavage of heme to biliverdin, carbon monoxide (CO), and ferrous iron, performs a cytoprotective function 49. Nevertheless, accumulating evidence indicates that excessive activation of HMOX1 exerts cytotoxic effects 50, possibly through the release of free iron and enhanced ferroptosis. For example, excessive induction of HMOX1 promotes cardiac ferroptosis and leads to cardiomyopathy in mice with sickle cell disease 51. Microglial HMOX1 overexpression contributes to neurotoxic iron accumulation and provides deleterious effects in aged mice exposed to inflammatory conditions 52. Besides, pharmacological inhibition or silencing of HMOX1 confers resistance to ferroptosis induced by Withaferin A and BAY 11-7085 53, 54. In this study, we found that HMOX1 is significantly elevated in RSL3-induced cell lines, mice models of ARDS, and RSL3- or LPS-challenged hAOs, accompanied by high levels of iron and lipid peroxidation. Furthermore, inhibition of HMOX1, through both pharmacologic and genetic approaches, relieved ferroptosis stimulated by RSL3 in lung epithelium and endothelium cells, while ectopic expression of HMOX1 attenuated the anti-ferroptotic effects of DIPY. Therefore, excessive activation of HMOX1 is likely a promoting factor for ferroptosis and ARDS. Supporting our findings, Weng and colleagues found that overexpressed HMOX1 in alveolar type II cells worsened oxidative stress accompanied by iron accumulation 55. In addition, total iron and non-heme iron levels in plasma and BALF were higher in ARDS patients than in healthy controls 10, 11. Therefore, targeting excessive HMOX1 to inhibit ferroptosis is a potential therapeutic strategy for treating ARDS. Whereas our study focused on HMOX1 expression in alveolar, airway, and lung endothelial cells, additional research is necessary to precisely delineate the differential contributions of HMOX1 in immune cells, like macrophages.

The regulation of HMOX1 expression has been extensively studied at the transcriptional level 56-58, at the post-transcription level 59, and at the protein degradation level 60, 61. In this study, we illustrated that activated CREB1 regulates the expression of HMOX1 at the transcription level in the context of ferroptosis in lung epithelial and endothelial cells. This finding is in line with previous studies which showed that HMOX1 is a direct transcriptional target of CREB1 62, 63. Specifically, we identified that CREB1 encourages the expression of HMOX1 mRNA through binding to the enhancer sites rather than the proximal promoter region (-2000 to +200) of the *HMOX1* gene. Further studies revealed that DIPY reduced CREB1 phosphorylation and then abolished the binding of CREB1 to the enhancers, thereby downregulating the expression of HMOX1. Moreover, through potential drug target screening and a series of validation methods, we demonstrated that DIPY directly binds to and activates SOD1, thereby inhibiting ferroptosis by blocking the CREB1/HMOX1 pathway. Our findings not only identified SOD1 as a novel and direct target of DIPY but also suggested that SOD1 acts as a negative regulator of CREB1 activity, at least in lung epithelial and endothelial cells. Consistent with our observations, it has been found that the overexpression of SOD1 reduced the phosphorylation of CREB1 in the liver of obese diabetic model mice 64. However, the precise mechanism by which SOD1 affects CREB1 requires further investigation.

Obtaining lung tissues from ARDS patients is difficult for ethical reasons. Given that the advantage of accurately recapitulate characteristics of *in vivo* tissues, lung organoids have emerged as valuable research tools for various respiratory diseases 65-68. In the previous study, we established hAOs through human embryonic stem cell (hESC)-induced differentiation and confirmed the critical role of the IL-13/SOCS1 pathway in ferroptosis of asthma 23. Generally, hESC-derived hAOs may not allow long-term expansion 21, and their generation and maintenance of hESC-derived hAOs are associated with high costs 69. Here, we successfully established adult human airway epithelial organoids by small amounts of normal lung tissue from NSCLC patients. These organoids showed a healthy, well-ciliated strip of respiratory epithelium, which mainly consists of ciliated cells and mucosecretory cells. We confirmed the anti-ferroptotic effect of DIPY using the patient-derived hAOs, consistent with findings observed in lung epithelial cells and ARDS mice models. Therefore, patient-derived hAOs represent an invaluable model for the study of lung diseases, offering significant insights into respiratory diseases. Despite advancements in generating patient-derived hAOs, airway organoids in our study may not fully replicate the clinical features of ARDS, as diffuse alveolar damage is one of the central and classic pathological features of the condition 2. We will further explore and optimize the culture of alveolar organoids and validation of the current data in the future.

In summary, in this study, we discovered that DIPY is a potent inhibitor of ferroptosis and holds promise as a therapeutic agent for treating ARDS. Moreover, our research elucidated that DIPY exerts its inhibitory effects on ferroptosis through the SOD1/CREB1/HMOX1 signaling pathway.

## Supplementary Material

Supplementary figures and tables.

Supplementary movie.

## Abbreviations

ALI: acute lung injury
AO/PI: acridine orange and propidium iodide staining
ARDS: acute respiratory distress syndrome
ATCC: American Type Culture Collection
BALF: bronchoalveolar lavage fluid
CCK-8: cell counting kit-8
CETSA: cellular thermal shift assay
CFUs: colony-forming units
ChIP: chromatin immunoprecipitation
CLP: cecal ligation and puncture
CREB1: cAMP responsive element binding protein 1
CRP: C-reactive protein
CT: computed tomography
DAB: diaminobenzidine
DARTS: drug affinity responsive target stability
DEGs: different expression genes
DIPY: dipyridamole
DMEM: Dulbecco's modified Eagle's medium
ELISA: enzyme-linked immunosorbent assay
FBS: fetal bovine serum
FDA: food and drug administration
GPX4: glutathione peroxidase 4
GSEA: gene set enrichment analysis
GSH: glutathione
hAOs: human airway organoids
H&E: hematoxylin/eosin
HMOX1: heme oxygenase 1
IF: immunofluorescence
IHC: immunohistochemical
LiP-MS: limited proteolysis-mass spectrometry
LPS: lipopolysaccharides
L-ROS: lipid reactive oxygen species
MDA: malondialdehyde
NSCLC: non-small cell lung cancer
PUFA: polyunsaturated fatty acid
qRT-PCR: quantitative real-time PCR
Rapa: rapamycin
RNA-Seq: RNA sequencing
RSL3: RAS-selective lethal 3
sgRNA: single guide RNA
shRNA: short hairpin RNA
siRNA: small interfering RNA
SOD1: superoxide dismutase 1
SOFA: sequential organ failure assessment
STS: staurosporine
TEM: transmission electron microscopy
W/D: wet/dry
Znpp: zinc protoporphyrin
