# Supplementary material for: Characterization of dipyridamole as a novel ferroptosis inhibitor and its therapeutic potential in acute respiratory distress syndrome management
Source: Theranostics. 2024 Oct 21;14(18):6947–68. doi: 10.7150/thno.102318 (PMC11610143; doi:10.7150/thno.102318)
Supplement: Supplementary file 1 — Supplementary figures and tables. [file thnov14p6947s1.pdf]

Supplementary Materials for

Characterization of dipyridamole as a novel ferroptosis inhibitor and its therapeutic potential in acute respiratory distress syndrome management

Xu Chen, Jiapan Shen, Xueqin Jiang, Min Pan, Shuang Chang, Juanjuan Li, Lei Wang, Manli Miao, Xiaoxia Feng, Ling Zhang, Guoqing Shu, Wenjian Liu, Fangzhou Xu, Wentao Zhang, Zhao Ding, Huaiyuan Zong, Weiwei Liu, Dapeng Li, Biao Chen, Min Shao, Guanghe Fei*, Xiaojun Zha*, Xiaoyun Fan*

Correspondence to: Xiaoyun Fan (xiaoyunfan@ahmu.edu.cn), Xiaojun Zha (zhaxiaojunpumc@gmail.com), and Guanghe Fei (guanghefei@126.com).

This PDF file includes:

Figure S1 to S11

Table S1 to S11

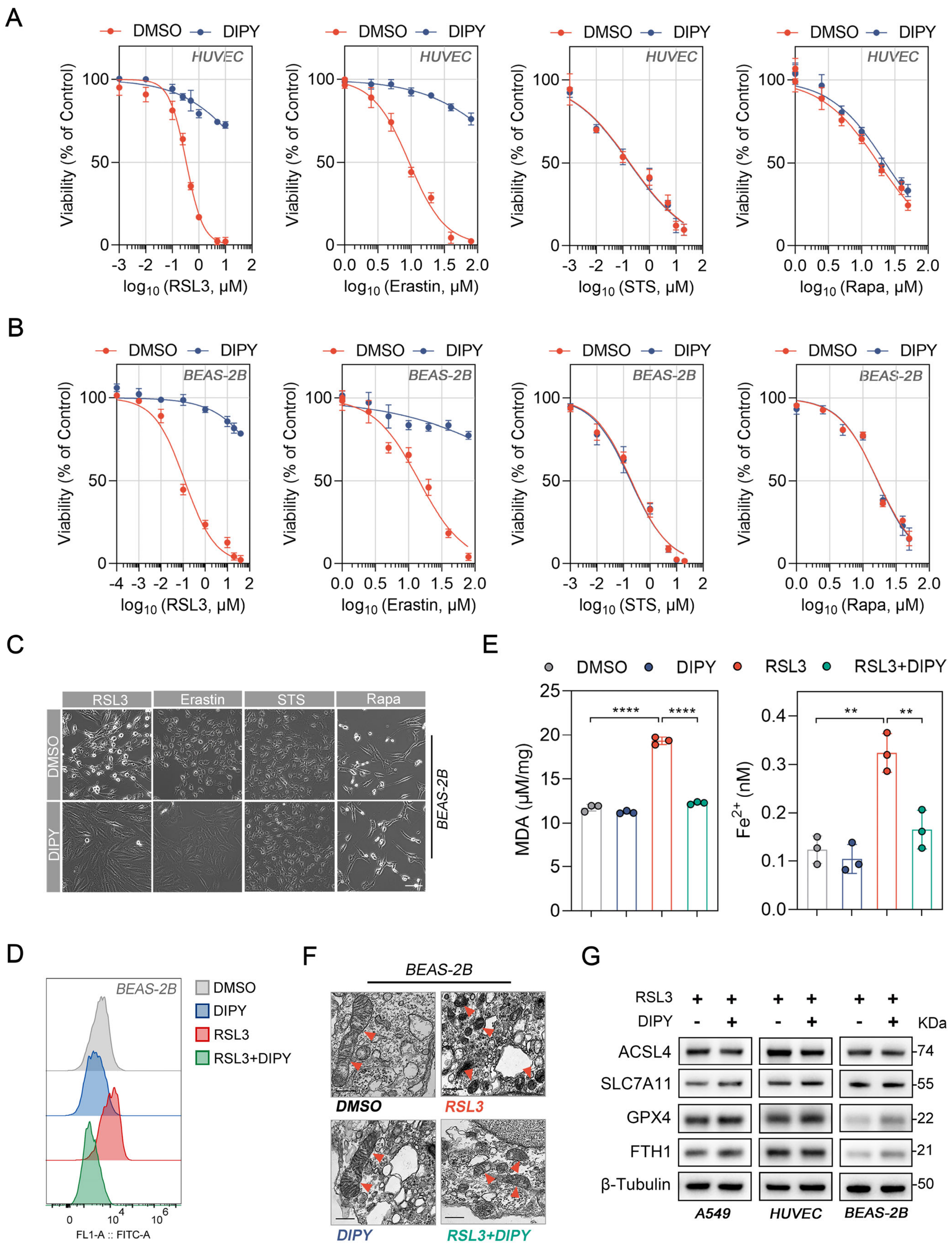
A
DMSO DIPY
HUVEC
Viability (% of Control)
log10 (RSL3, μM)
log10 (Erastin, μM)
log10 (STS, μM)
log10 (Rapa, μM)
B
BEAS-2B
C
RSL3
Erastin
STS
Rapa
DMSO
DIPY
BEAS-2B
E
DMSO DIPY RSL3 RSL3+DIPY
MDA (μM/mg)
Fe2+ (nM)
****
**
D
BEAS-2B
DMSO
DIPY
RSL3
RSL3+DIPY
FL1-A :: FITC-A
F
BEAS-2B
DMSO
RSL3
DIPY
RSL3+DIPY
G
RSL3
DIPY
KDa
ACSL4
SLC7A11
GPX4
FTH1
β-Tubulin
-74
-55
-22
-21
-50
A549
HUVEC
BEAS-2B

Figure. S1 Validation of the specific anti-ferroptosis effect of DIPY in lung epithelial and endothelial cells.

(**A-C**) HUVEC (**A**) and BEAS-2B (**B**) cells were treated with RSL3, Erastin, STS, and Rapa with or without DIPY (5 μM for HUVEC; 10 μM for BEAS-2B) for indicated times (RSL3 and Erastin for 8 h; STS for 6 h; Rapa for 48 h). CCK-8 assay for cell viability. Representative phase-contrast images of BEAS-2B cells (**C**). Scale bar, 100 μm. (**D-F**) BEAS-2B cells were treated with RSL3 (1 μM), DIPY (10 μM), or RSL3 (1 μM) plus DIPY (10 μM) for 8 h. L-ROS levels were measured (**D**). MDA contents and Fe^{2+} levels were determined (**E**). Representative TEM images of cells were presented, with red arrows indicating mitochondria (**F**). The expression of ferroptosis-related markers was detected by western blot (**G**). Scale bar, 500 nm. Results are shown as mean ± SD from 3 independent experiments. Statistical significance is indicated as $^{**}P < 0.01$; $^{****}P < 0.0001$.

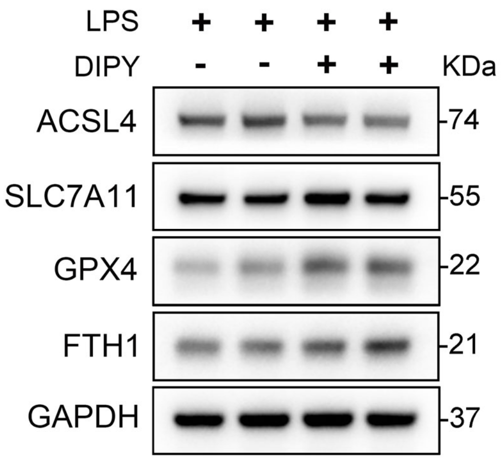

Figure. S2 The expression of ferroptosis marker proteins in LPS-induced ARDS mouse model.

Western blot assays examined the expression of ferroptosis marker proteins (ACSL4, SLC7A11, GPX4, and FTH1) in LPS-induced acute lung injury mouse models with or without DIPY treatment as shown in Figure 2 (n = 6 per group).

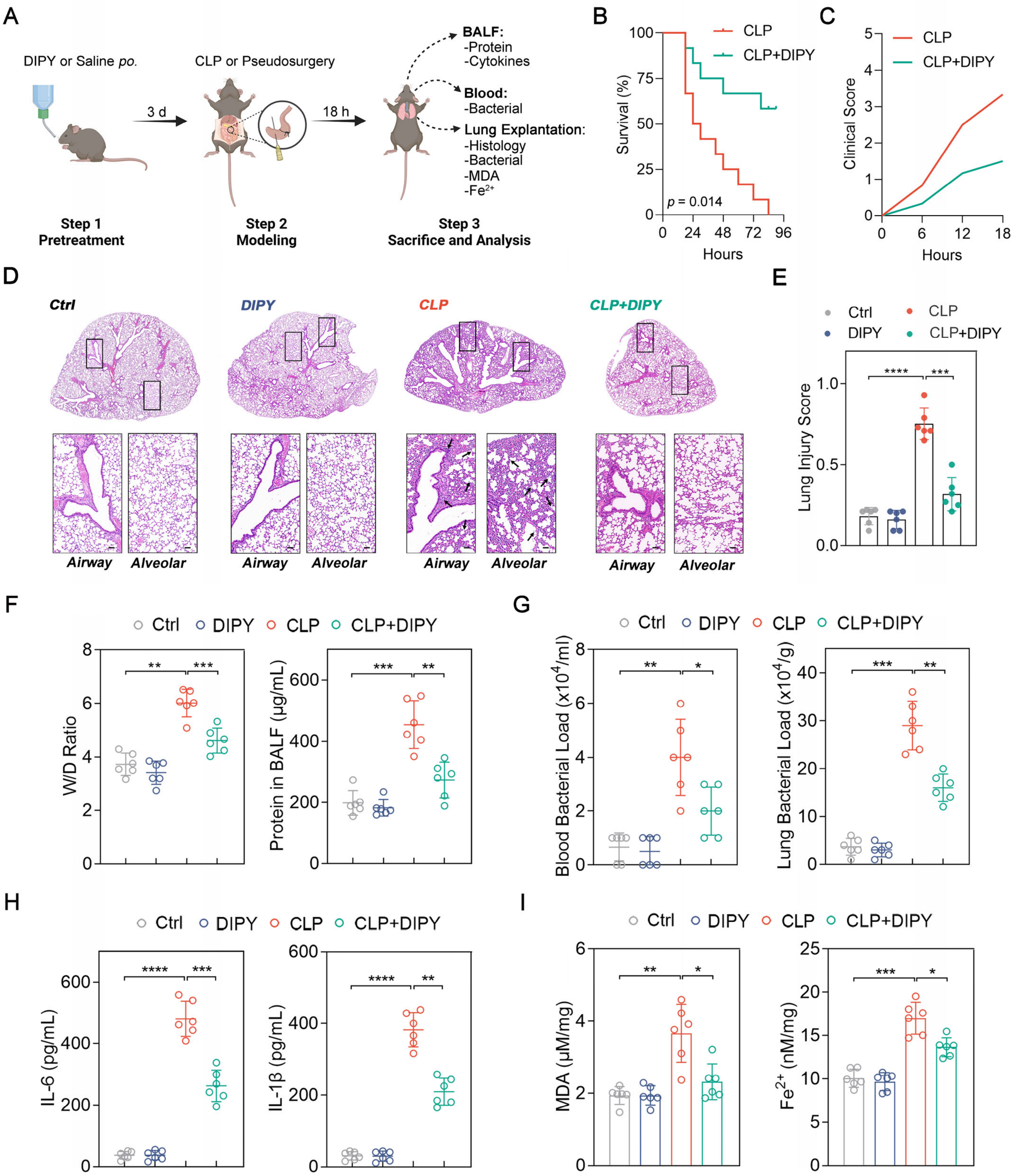
A
DIPY or Saline po.
3 d
CLP or Pseudosurgery
18 h
BALF:
-Protein
-Cytokines
Blood:
-Bacterial
Lung Explantation:
-Histology
-Bacterial
-MDA
-Fe2+
Step 1
Pretreatment
Step 2
Modeling
Step 3
Sacrifice and Analysis
B
CLP
CLP+DIPY
Survival (%)
p = 0.014
Hours
C
Clinical Score
Hours
D
Ctrl
DIPY
CLP
CLP+DIPY
Airway
Alveolar
E
Lung Injury Score
F
W/D Ratio
Protein in BALF (μg/mL)
G
Blood Bacterial Load (x10^4/ml)
Lung Bacterial Load (x10^4/g)
H
IL-6 (pg/mL)
IL-1β (pg/mL)
I
MDA (μM/mg)
Fe2+ (nM/mg)

Figure. S3 DIPY inhibits ferroptosis in CLP-induced sepsis mouse model.

(**A**) Scheme of the experimental procedure for the CLP-induced sepsis mouse model. DIPY pretreatment (10 mg/kg, orally) was administered 3 days before the CLP surgery. Mice underwent either mild CLP (ligated ~50% cecum) or lethal CLP (ligated ~75% cecum). After 18 h, the BALF and lung samples from mild CLP mice were collected and analyzed. (**B**) Percent survival after lethal CLP is shown (n = 12 per group). (**C-I**) Mild CLP-challenged sepsis mice were pretreated with or without DIPY (n = 6 per group). Clinical score of mice (**C**). Representative images of H&E-stained lung sections, with black arrows indicating infiltrating inflammatory cells, interstitial edema, and alveolar wall thickening (**D**). Scale bar, 50 μm. Lung injury scores (**E**). Measurements of lung wet/dry ratio and total protein in BALF (**F**). Bacterial load in the blood and lungs (**G**). ELISA analysis of IL-6 and IL-1β levels in BALF (**H**). MDA contents and Fe^{2+} levels in mouse lung tissues (**I**). Results are shown as mean ± SD. Statistical significance is indicated as *$P < 0.05$; **$P < 0.01$; ***$P < 0.001$; ****$P < 0.0001$.

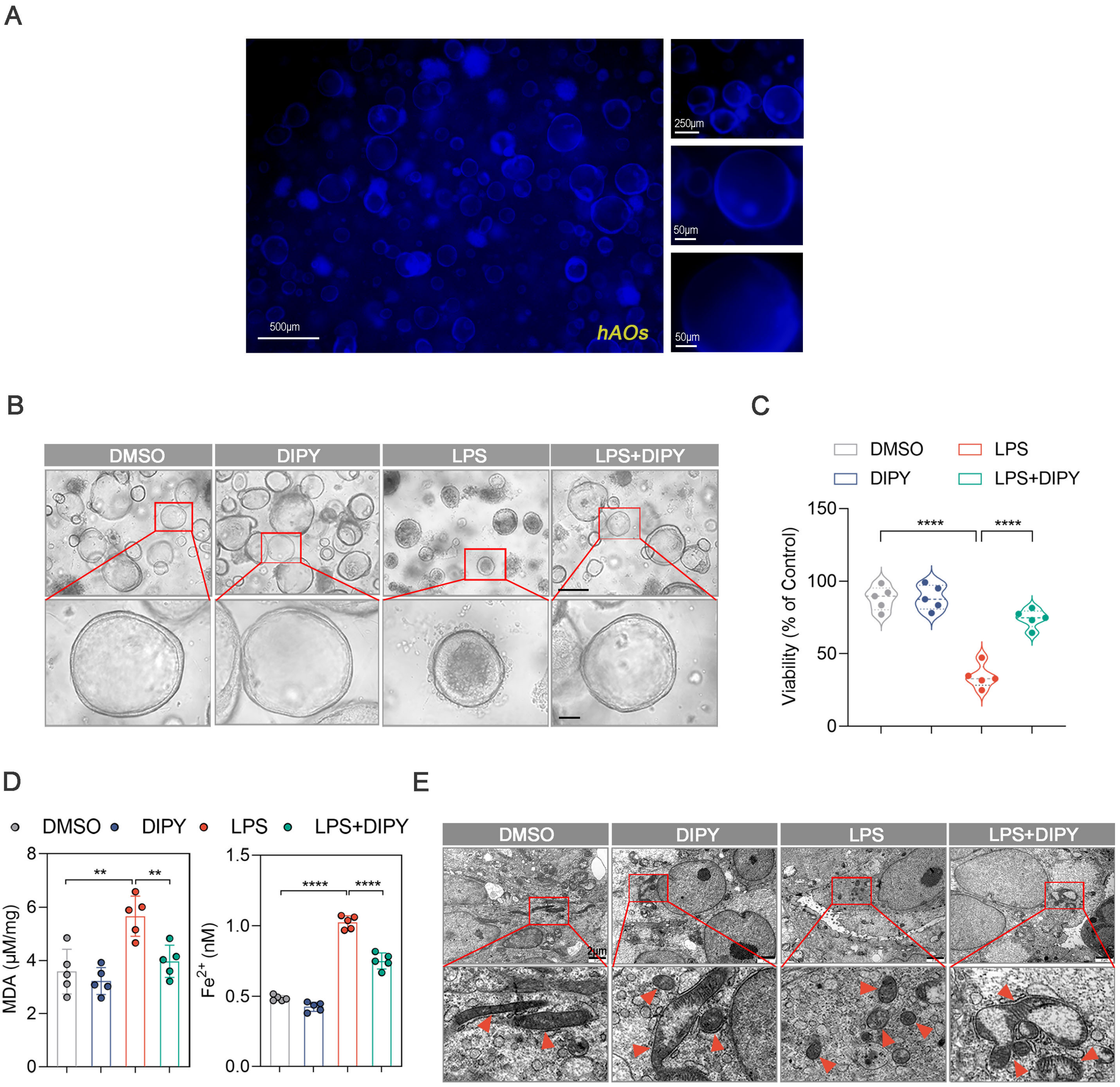

A
500μm
hAOs
250μm
50μm
50μm
B
DMSO
DIPY
LPS
LPS+DIPY
C
DMSO
LPS
DIPY
LPS+DIPY
Viability (% of Control)
150
100
50
0
****
****
D
DMSO
DIPY
LPS
LPS+DIPY
MDA (μM/mg)
8
6
4
2
0
**
**
Fe2+ (nM)
1.5
1.0
0.5
0.0
****
****
E
DMSO
DIPY
LPS
LPS+DIPY
2μm

Figure. S4 DIPY inhibited LPS-induced ferroptosis in hAOs.

(**A**) hAOs were treated with DIPY (20 μM) for 24 h and observed by confocal microscopy with excitation at 405 nm. (**B-E**) The hAOs were treated with LPS (100 μg/mL), DIPY (20 μM), and LPS (100 μg/mL) with DIPY (20 μM) for 24 h (n = 5 per group). Morphological changes of hAOs after the indicated treatment (**B**). Scale bar, 250 μm (upper panel) and 50 μm (lower panel). Assessment of organoid viability (**C**). MDA contents and Fe^{2+} levels were measured (**D**). TEM analyses illustrating representative mitochondria in the hAOs with red arrows indicate mitochondria (**E**). Scale bar, 2 μm. Results are shown as mean ± SD. Statistical significance is indicated as $^{**}P < 0.01$; $^{****}P < 0.0001$.

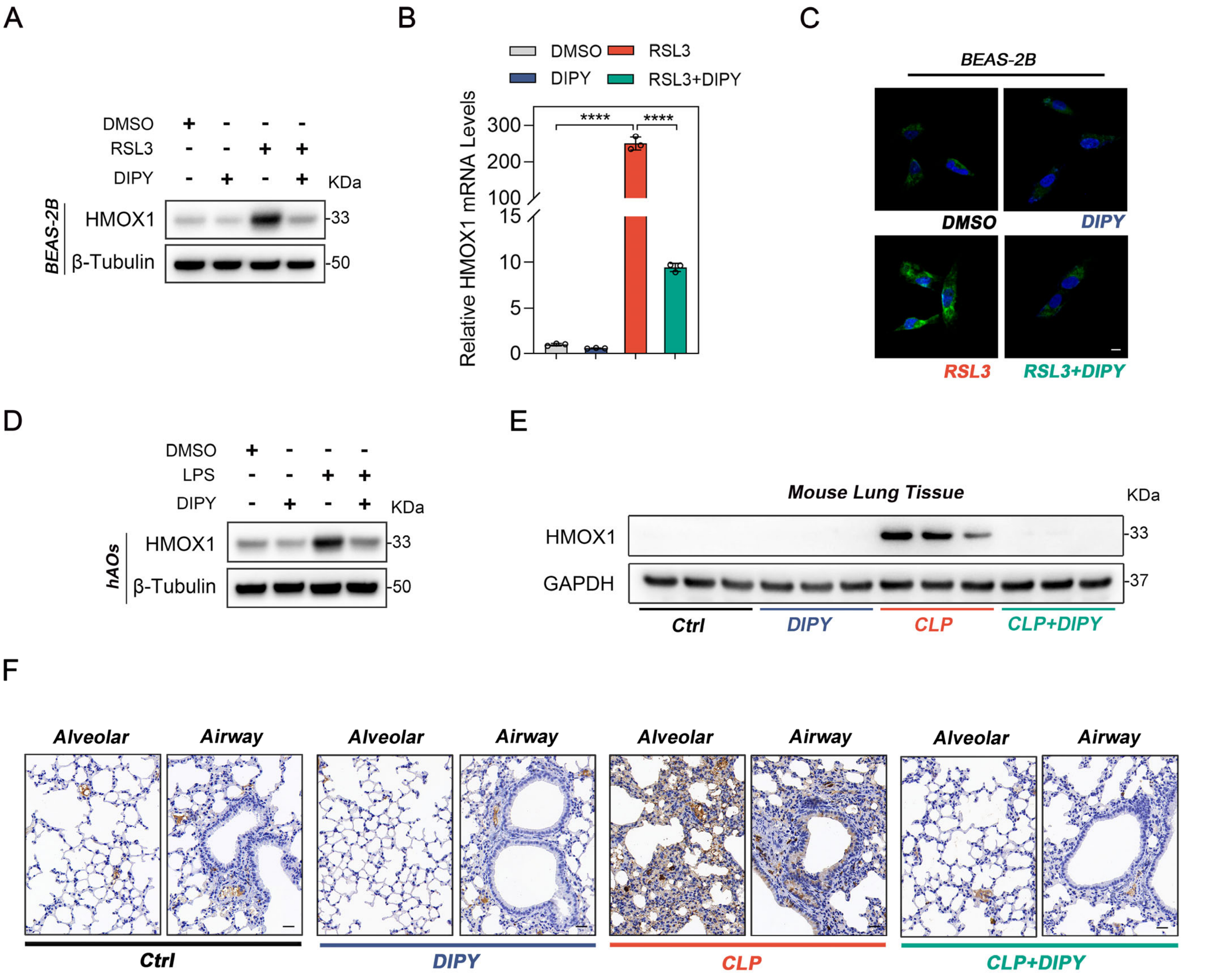

Figure. S5 Validation of HMOX1 as a target of DIPY.

(**A-C**) BEAS-2B cells were treated with RSL3, DIPY, or RSL3 plus DIPY for 8 h as shown in Figure S1 (n = 3 per group). The expression of the HMOX1 was detected by western blot (**A**), qRT-PCR (**B**), and IF staining (**C**). Scale bar, 10 µm. (**D**) hAOs were treated with LPS, DIPY, and LPS with DIPY for 24 h as shown in Figure S4, and the protein levels of HMOX1 were analyzed by western blot (n = 5 per group). (**E** and **F**) The expression of HMOX1 in CLP-induced sepsis mouse models with or without DIPY treatment was detected by western blot (**E**) and IHC (**F**) assays (n = 6 per group). Scale bar, 50 µm. Results are shown as mean ± SD of 3-6 independent experiments. Statistical significance is indicated as $****P < 0.0001$.

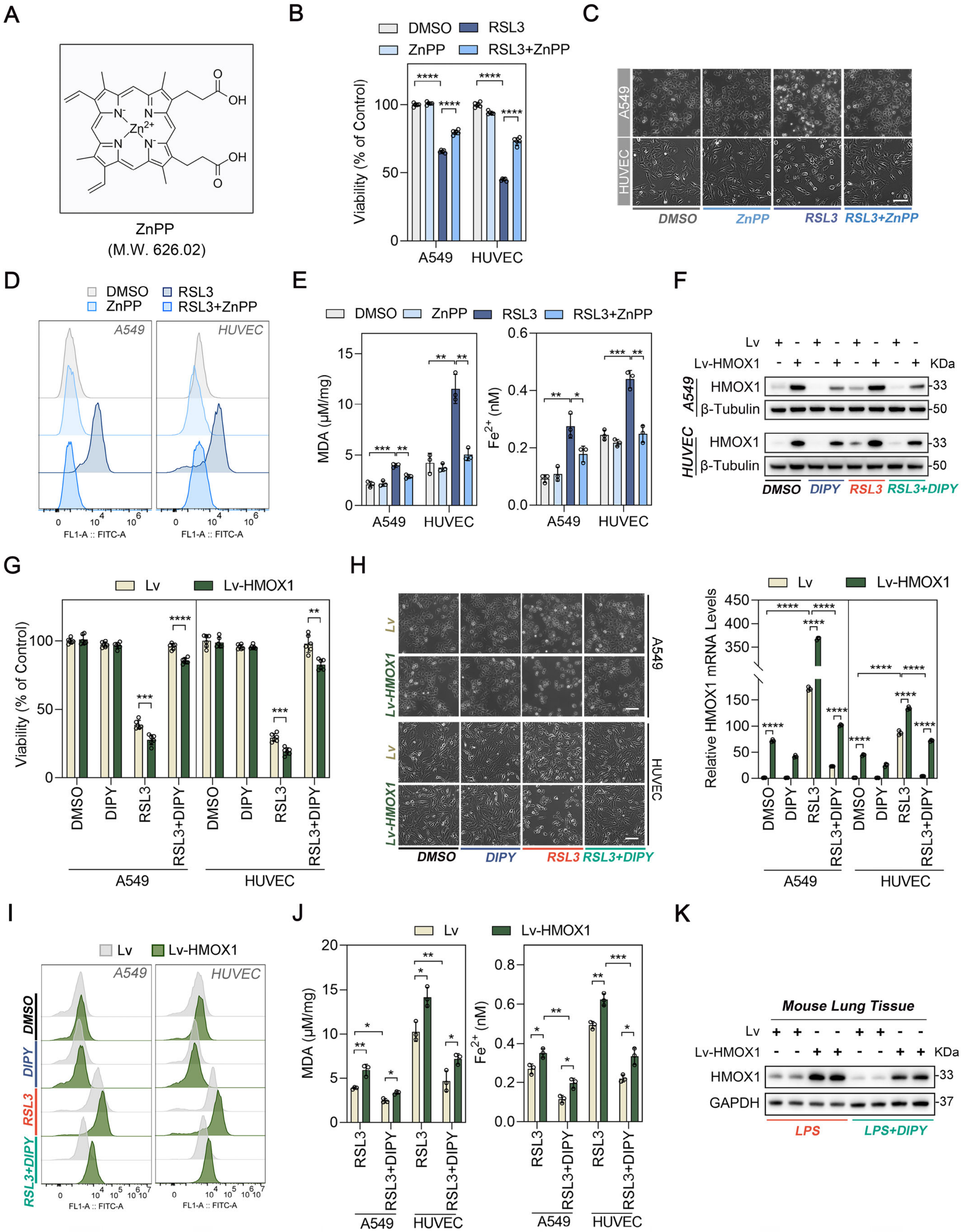
A
ZnPP
(M.W. 626.02)
B
DMSO
RSL3
ZnPP
RSL3+ZnPP
Viability (% of Control)
A549
HUVEC
C
A549
HUVEC
DMSO
ZnPP
RSL3
RSL3+ZnPP
D
A549
HUVEC
FL1-A :: FITC-A
E
MDA (μM/mg)
Fe2+ (nM)
F
Lv
Lv-HMOX1
KDa
HMOX1
β-Tubulin
DMSO
DIPY
RSL3
RSL3+DIPY
G
Lv
Lv-HMOX1
Viability (% of Control)
H
Relative HMOX1 mRNA Levels
I
Lv
Lv-HMOX1
FL1-A :: FITC-A
J
MDA (μM/mg)
Fe2+ (nM)
K
Mouse Lung Tissue
HMOX1
GAPDH
LPS
LPS+DIPY

Figure. S6 DIPY inhibits ferroptosis by downregulating HMOX1.

(**A**) The chemical structure of ZnPP is shown. (**B-E**) A549 were treated with RSL3 (5 μM), ZnPP (5 μM), and RSL3 (5 μM) plus ZnPP (5 μM) for 8 h (n = 3 per group). HUVEC cells were treated with RSL3 (0.25 μM), ZnPP (5 μM), and RSL3 (0.25 μM) plus ZnPP (5 μM) for 8 h (n = 3 per group). Cell viability was assessed (**B**). Representative phase-contrast images of cells (**C**). Scale bar, 100 μm. Measurement of L-ROS levels (**D**). Measurement of MDA contents and Fe^{2+} levels (**E**). (**F-J**) A549 and HUVEC cells were infected with Lv-HMOX1 or Lv. Lv-HMOX1 and Lv A549 cells were treated by RSL3 (5 μM), DIPY (10 μM), and RSL3 (5 μM) plus DIPY (10 μM; n = 3 per group). Lv-HMOX1 and Lv HUVEC cells were treated by RSL3 (0.25 μM), DIPY (5 μM), and RSL3 (0.25 μM) plus DIPY (5 μM; n = 3 per group). The expression of HMOX1 was confirmed by western blot (upper panel of **F**) and qRT-PCR (lower panel of **F**). Cell viability assessment (**G**). Representative phase-contrast images of cells (**H**). Scale bar, 100 μm. Measurement of L-ROS levels (**I**). MDA contents and Fe^{2+} levels (**J**). (**K**) C57/BL6 mice were administered Lv-HMOX1 or Lv via intratracheal instillation for 7 days. On day 8, the indicated mice were challenged with LPS (2.5 mg/kg) with or without DIPY pretreatment as described in Figure 5I-M. The expression of HMOX1 was detected by western blot (n = 6 per group). Results are shown as mean ± SD of at least 3 independent experiments. Statistical significance is indicated as $^{*}P < 0.05$; $^{**}P < 0.01$; $^{***}P < 0.001$; $^{****}P < 0.0001$.

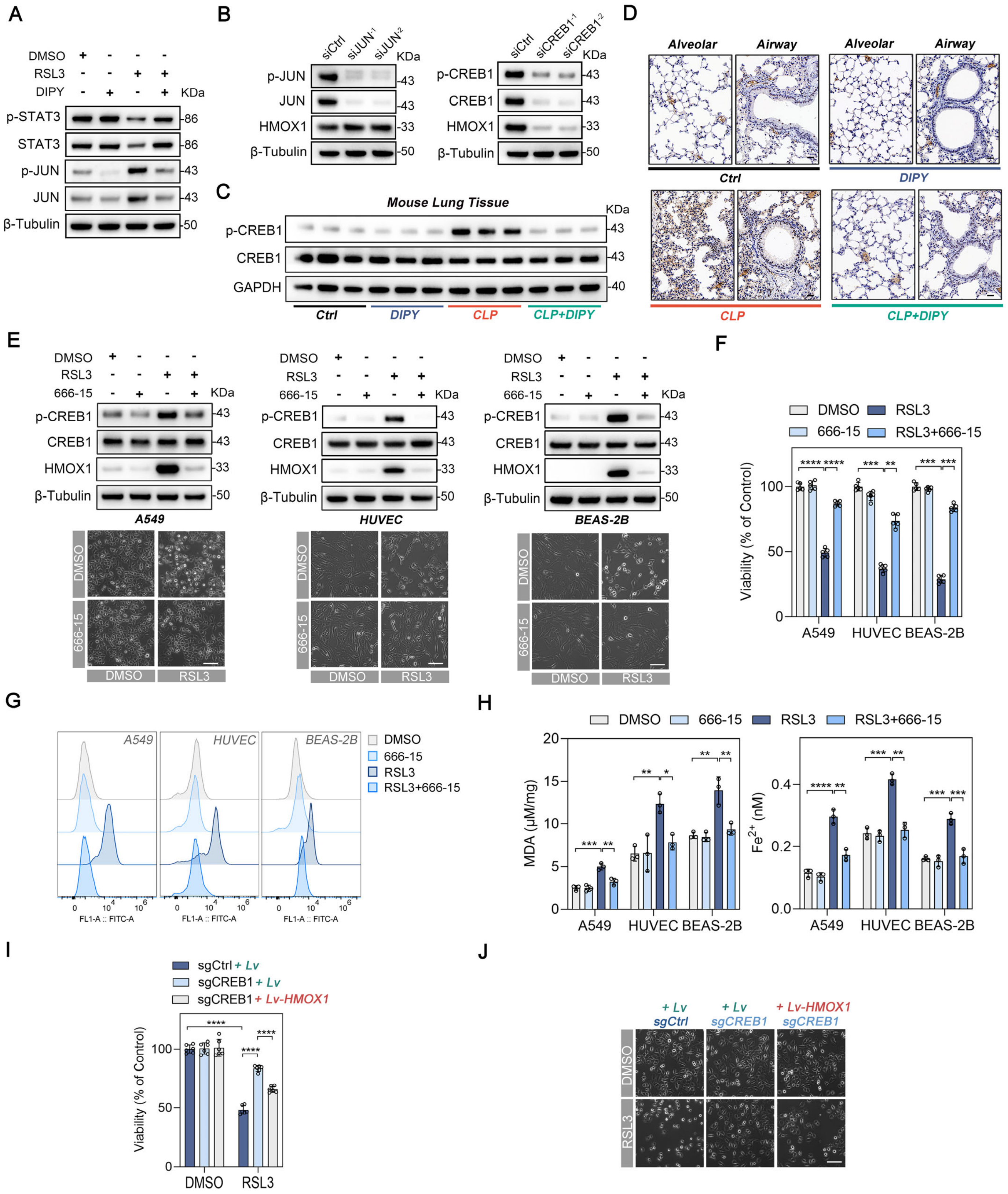
A
DMSO
RSL3
DIPY
KDa
p-STAT3
STAT3
p-JUN
JUN
β-Tubulin
B
siCtrl
siJUN-1
siJUN-2
p-JUN
JUN
HMOX1
β-Tubulin
siCREB1-1
siCREB1-2
p-CREB1
CREB1
C
Mouse Lung Tissue
GAPDH
Ctrl
DIPY
CLP
CLP+DIPY
D
Alveolar
Airway
E
666-15
A549
HUVEC
BEAS-2B
F
RSL3+666-15
Viability (% of Control)
G
FL1-A :: FITC-A
H
MDA (μM/mg)
Fe2+ (nM)
I
sgCtrl + Lv
sgCREB1 + Lv
sgCREB1 + Lv-HMOX1
J

Figure. S7 DIPY inhibits HMOX1 by deactivating CREB1.

(**A**) A549 cells were treated with RSL3, DIPY, and RSL3 with DIPY for 8 h as shown in Figure 1. Cell lysates were then subjected to immunoblotting with the indicated antibodies. (**B**) A549 were transfected with JUN siRNAs ($siJUN^{-1}$, $siJUN^{-2}$), CREB1 siRNAs ($siCREB1^{-1}$ and $siCREB1^{-2}$), or control siRNA (siCtrl) for 48 h. Cell lysates were subjected to immunoblotting with the indicated antibodies. (**C-D**) Western blot assays (**C**) and IHC assays (**D**) examined the expression of p-CREB1 in CLP-induced sepsis mouse models with or without DIPY treatment as shown in Figure S3 (n = 6 per group). Scale bar, 100 µm. (**E-H**) A549, HUVEC, and BEAS-2B cells were treated by RSL3 (5 µM for A549, 0.25 µM for HUVEC, and 1 µM for BEAS-2B), 666-15 (1 µM), and RSL3 (5 µM for A549, 0.25 µM for HUVEC, and 1 µM for BEAS-2B) with 666-15 (1 µM) for 8 h. Cell lysates were subjected to immunoblotting with the indicated antibodies (up panels of **E**). Representative phase-contrast images of cells (lower panels of **E**). Scale bar: 100 µm. Cell viability assessment (**F**). Measurement of L-ROS levels (**G**). Determination of MDA contents and Fe^{2+} levels (**H**). (**I-J**) sgCREB1 A549 cells or the control cells were infected with lentiviruses carrying Lv-HMOX1 or Lv and then treated with RSL3 as shown in Figure 6. Cell viability assessment (**I**). Representative phase-contrast images of indicated cells (**J**). Scale bar, 100 µm. Results are shown as mean ± SD of 3-6 independent experiments. Statistical significance is indicated as $*P < 0.05$; $**P < 0.01$; $***P < 0.001$; $****P < 0.0001$.

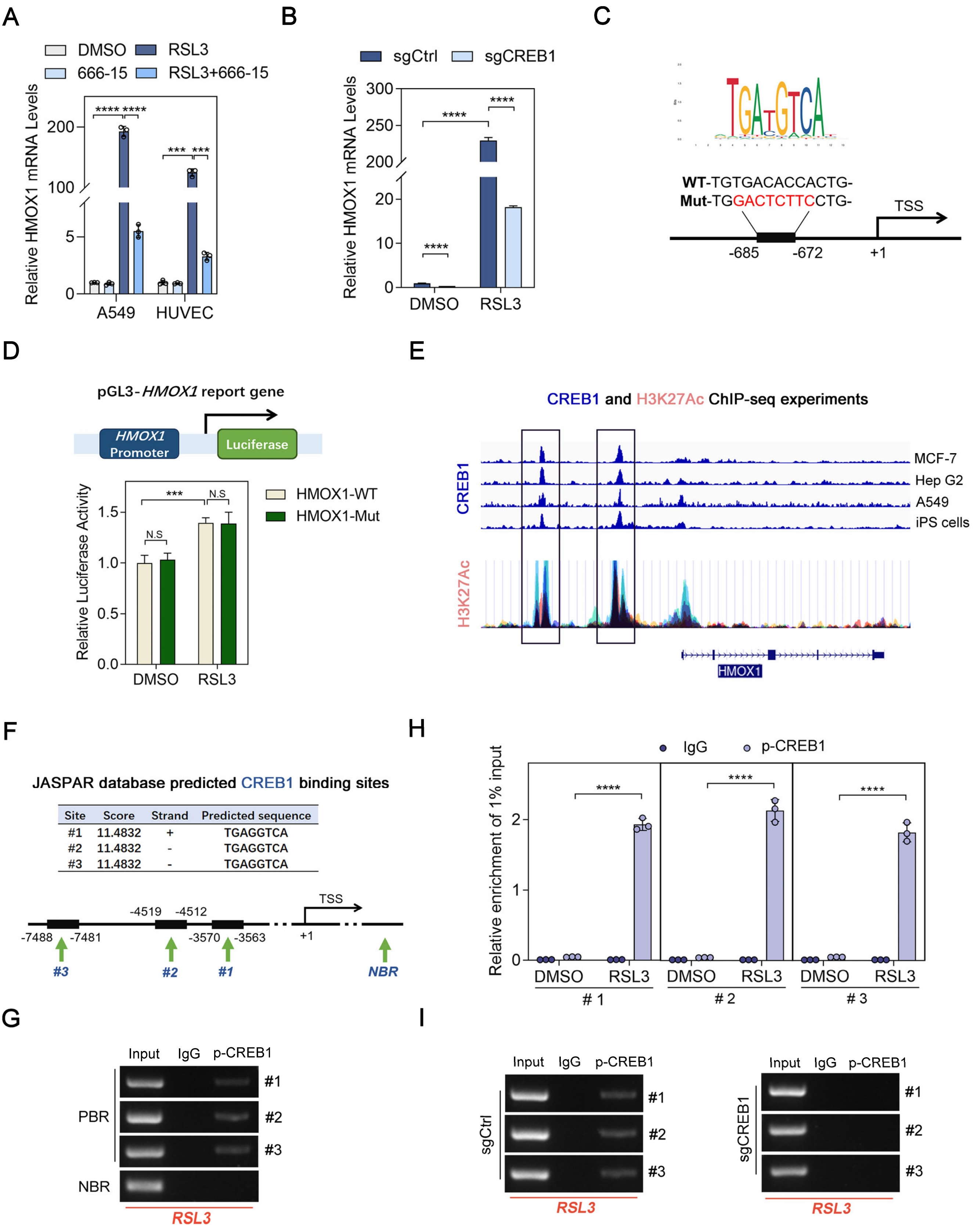

A
DMSO
RSL3
666-15
RSL3+666-15
Relative HMOX1 mRNA Levels
A549
HUVEC
B
sgCtrl
sgCREB1
Relative HMOX1 mRNA Levels
DMSO
RSL3
C
WT-TGTGACACCACTG-
Mut-TGGACTCTTCCTG-
TSS
-685
-672
+1
D
pGL3-HMOX1 report gene
HMOX1 Promoter
Luciferase
Relative Luciferase Activity
HMOX1-WT
HMOX1-Mut
N.S
DMSO
RSL3
E
CREB1 and H3K27Ac ChIP-seq experiments
CREB1
H3K27Ac
MCF-7
Hep G2
A549
iPS cells
HMOX1
F
JASPAR database predicted CREB1 binding sites
Site Score Strand Predicted sequence
#1 11.4832 + TGAGGTCA
#2 11.4832 - TGAGGTCA
#3 11.4832 - TGAGGTCA
TSS
-4519 -4512
-7488 -7481
-3570 -3563
+1
#3
#2
#1
NBR
G
Input IgG p-CREB1
PBR
NBR
#1
#2
#3
RSL3
H
IgG
p-CREB1
Relative enrichment of 1% input
DMSO RSL3 DMSO RSL3 DMSO RSL3
1
2
3
I
Input IgG p-CREB1
sgCtrl
sgCREB1
#1
#2
#3
RSL3

Figure. S8 CREB1 transcriptionally upregulates the expression of HMOX1 through binding to the enhancers.

(**A**) A549 and HUVEC cells were treated with RSL3, 666-15, and RSL3 plus 666-15 for 8 h as shown in Figure S7. Total RNA was extracted, and qRT-PCR was performed to detect the mRNA levels of HMOX1. (**B**) sgCREB1 A549 cells were treated with RSL3 for 8 h as shown in Figure 6. The level of HMOX1 mRNA was analyzed by qRT-PCR. (**C**) Schematic representation of the putative CREB1-binding site in the promoter of the human *HMOX1* gene. (**D**) The human *HMOX1* promoter regions (-847 to +65), with or without the putative CREB1-binding motif mutation, were cloned into pGL3-basic reporter plasmids and transfected into A549 cells for 24 h. These cells were treated with RSL3 (5 μM) or DMSO for 8 h, and the relative luciferase activity was determined. (**E**) Analysis of CREB1-binding peaks (upper panel) and the active enhancer marker H3K27Ac peaks (lower panel) upstream of the *HMOX1* promoter using ChIP-Atlas and ENCODE databases. (**F**) Prediction of CREB1 binding sites in the regions around the CREB1-binding peaks shown in E using the JASPAR database. (**G**) A549 cells were treated with RSL3 (5 μM) for 8 h. Analysis of the enrichment of CREB1 in the three potential CREB1-binding sites by ChIP-PCR assay. (**H**) A549 cells were subjected to ChIP analysis with antibodies to p-CREB1 or control rabbit IgG. qRT-PCR was performed to amplify regions surrounding the putative CREB1 binding sites (#1, #2, and #3). Data are plotted as the ratio of immunoprecipitated DNA to total input DNA. (**I**) The sgCREB1 and sgCtrl A549 cells were treated with RSL3 (5 μM), and the enrichment of CREB1 in the three sites was analyzed by ChIP-PCR assay. Results are shown as mean ± SD of 3 independent experiments. Statistical significance is indicated as ***$P < 0.001$; ****$P < 0.0001$; N.S. not significant.

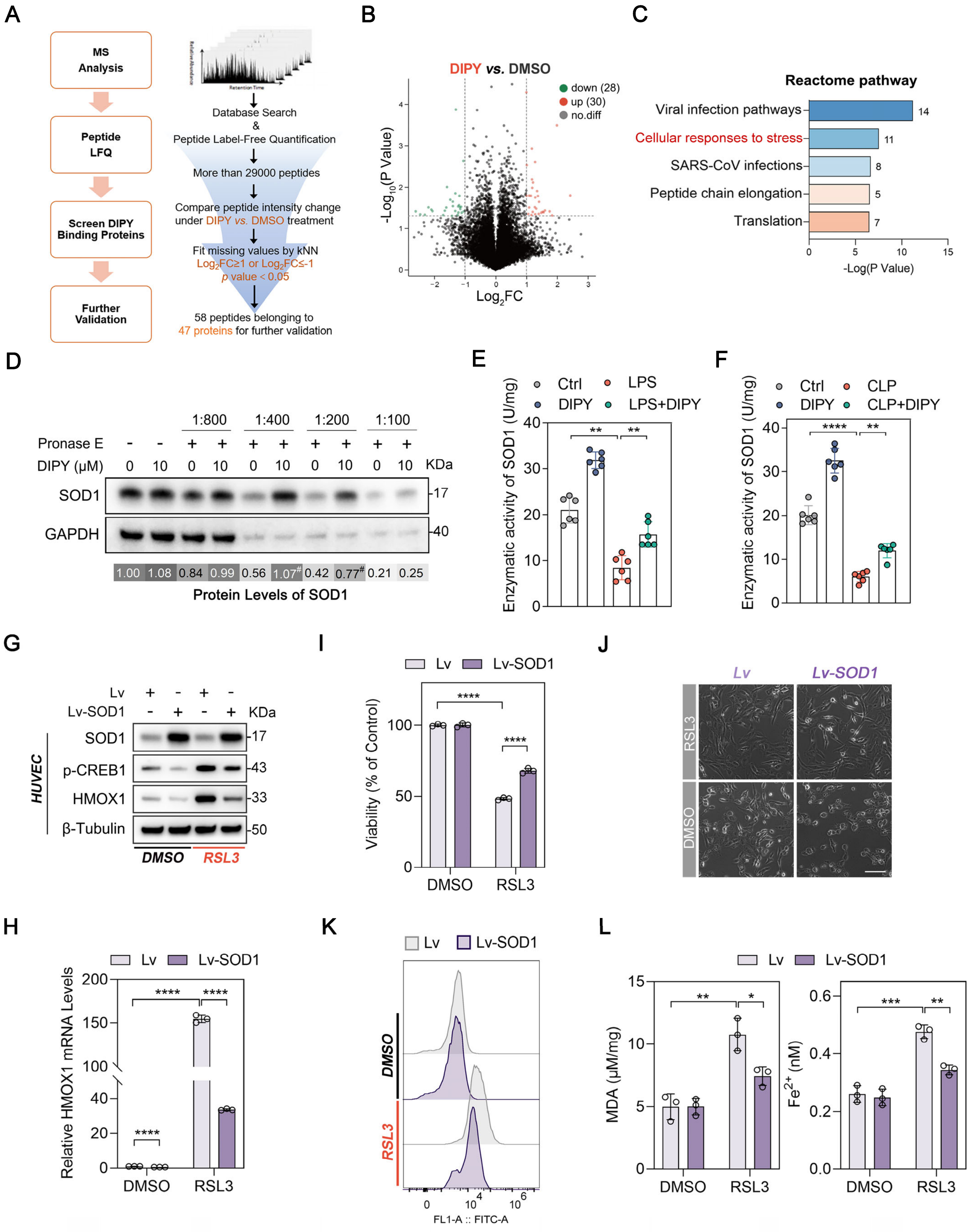

A
MS Analysis
Peptide LFQ
Screen DIPY Binding Proteins
Further Validation
Database Search & Peptide Label-Free Quantification
More than 29000 peptides
Compare peptide intensity change under DIPY vs. DMSO treatment
Fit missing values by kNN
Log2FC≥1 or Log2FC≤-1
p value < 0.05
58 peptides belonging to 47 proteins for further validation
B
DIPY vs. DMSO
down (28)
up (30)
no.diff
-Log10(P Value)
Log2FC
C
Reactome pathway
Viral infection pathways
Cellular responses to stress
SARS-CoV infections
Peptide chain elongation
Translation
-Log(P Value)
D
1:800
1:400
1:200
1:100
Pronase E
DIPY (μM)
KDa
SOD1
GAPDH
1.00 1.08 0.84 0.99 0.56 1.07# 0.42 0.77# 0.21 0.25
Protein Levels of SOD1
E
Ctrl
LPS
DIPY
LPS+DIPY
Enzymatic activity of SOD1 (U/mg)
F
Ctrl
CLP
DIPY
CLP+DIPY
Enzymatic activity of SOD1 (U/mg)
G
Lv
Lv-SOD1
KDa
HUVEC
SOD1
p-CREB1
HMOX1
β-Tubulin
DMSO
RSL3
I
Lv
Lv-SOD1
Viability (% of Control)
DMSO
RSL3
J
Lv
Lv-SOD1
RSL3
DMSO
H
Lv
Lv-SOD1
Relative HMOX1 mRNA Levels
DMSO
RSL3
K
Lv
Lv-SOD1
DMSO
RSL3
FL1-A :: FITC-A
L
Lv
Lv-SOD1
MDA (μM/mg)
Fe2+ (nM)
DMSO
RSL3

Figure. S9 DIPY suppresses the CREB1/HMOX1 pathway by directly binding to and activating SOD1.

(**A**) Computational pipeline for screening of peptides corresponding to DIPY binding proteins. (**B**) Visualization of LiP-MS assay results using volcano plots. (**C**) Reactome pathway analysis of candidate DIPY target proteins identified by the LiP-MS assay. (**D**) Verification of SOD1 as a target of DIPY using the DARTS assay. (**E**) Enzymatic activity of SOD1 in LPS-induced ALI mouse models with or without DIPY treatment. (**F**) Enzymatic activity of SOD1 in CLP-induced sepsis mouse models with or without DIPY treatment. (**G-L**) HUVEC cells were infected with lentivirus harboring a vector encoding SOD1 (Lv-SOD1) or the empty vector (Lv). The indicated cells were treated with or without RSL3 (0.25 μM). Cell lysates were subjected to immunoblotting with the indicated antibodies (**G**). The expression of HMOX1 mRNA was analyzed using qRT-PCR (**H**). Assessment of cell viability (**I**). Representative phase-contrast images of cells (**J**). Scale bar, 100 μm. Measurement of L-ROS levels (**K**). Determination of MDA contents and Fe^{2+} levels (**L**). Results are shown as mean ± SD of 3-6 independent experiments. Statistical significance is indicated as *$P < 0.05$; **$P < 0.01$; ***$P < 0.001$; ****$P < 0.0001$.

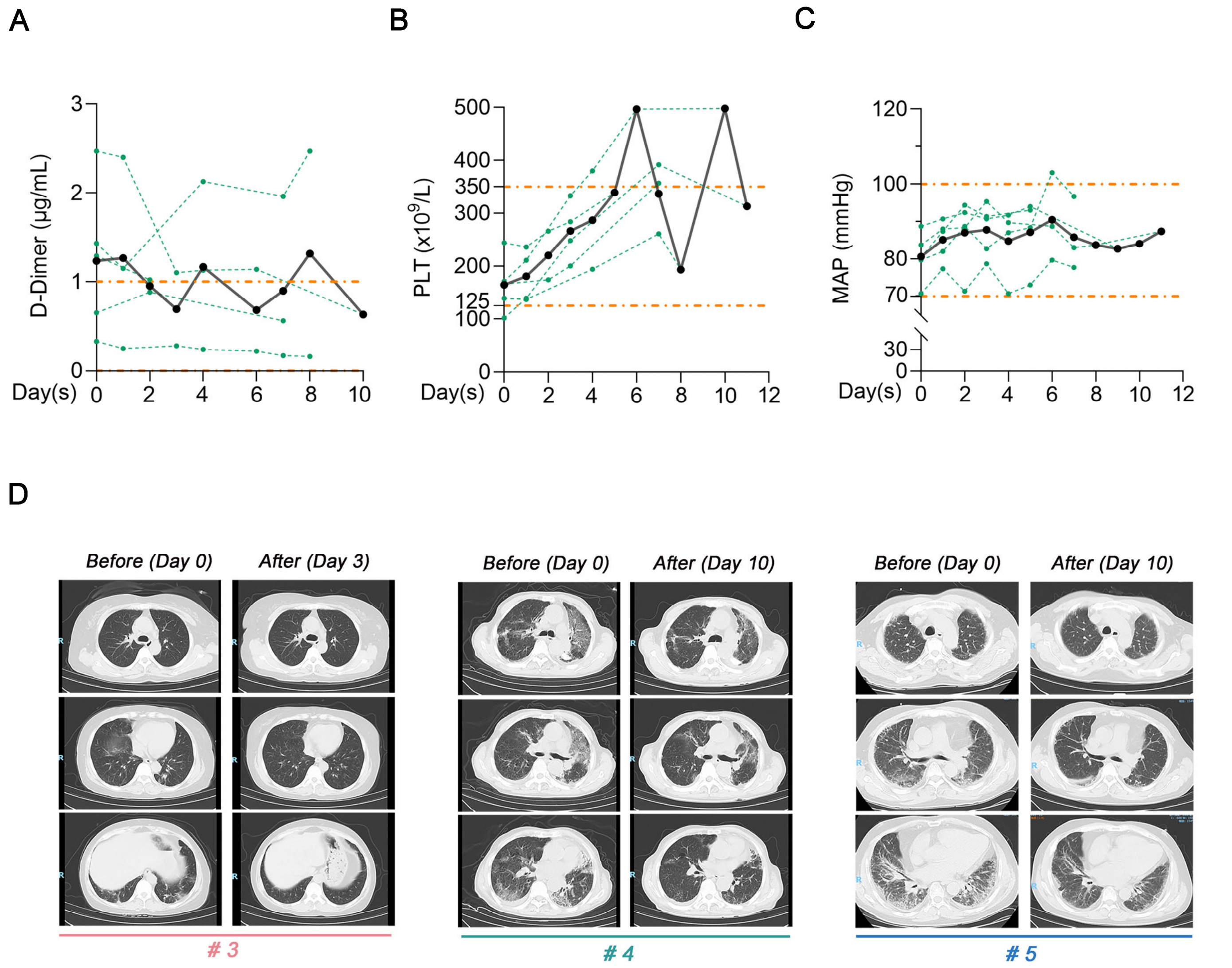

Figure. S10 Coagulation function, MAP, and chest CT of subjects after DIPY treatment.

(**A**) D-Dimer levels at indicated time points before and after DIPY treatment. (**B**) Peripheral blood platelet counts at indicated time points before and after DIPY treatment. (**C**) The mean arterial pressure (MAP) at indicated time points before and after DIPY treatment. (**A-C**) Data is shown with individual patient responses as dotted lines and the mean of all subjects as solid lines. (**D**) Chest computed tomography scans of three patients (#3, #4, and #5) before DIPY treatment (Day 0) and after DIPY treatment (Day 3 or 10).

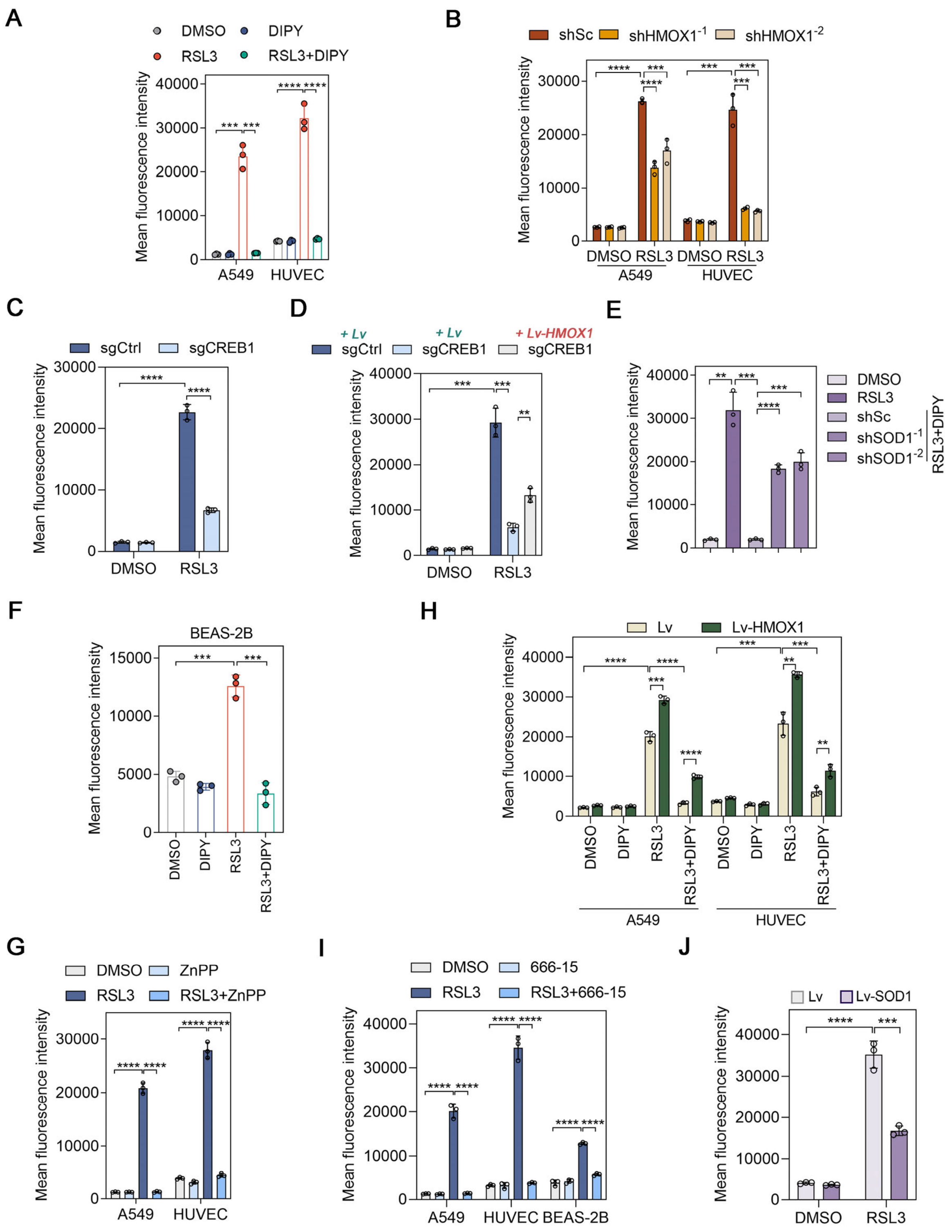

Figure. S11 The quantification analysis of the flow cytometric assays.

(**A-J**) The quantitative analysis results of flow cytometric assays in Figures 1G, 5D, 6J, 6M, and 7K, and Supplemental Figures S1D, S6D, S6I, S7G, and S9K are presented in A, B, C, D, E, F, G, H, I, and J respectively.

Table. S1 Identified three TF candidates for the human *HMOX1* gene.

TFs predicated by four TF databases [A] (14)	TFs analyzed by Metascape [B] (20)	TFs candidates for *HMOX1* (3)
STAT1	ETS1	CREB1
STAT3	HIF1A	STAT3
KLF9	NFKB1	JUN
TEAD4	SP1	
MYC	RELA	
TFAP2C	HSF1	
JUN	JUN	
CTCF	JUND	
GATA2	PPARA	
YY1	PPARG	
FOXA1	FLI1	
AR	TFAP2A	
CREB1	STAT3	
MAZ	SP3	
	SREBF1	
	CREB1	
	ETV4	
	VDR	
	SIRT1	
	KLF6	

[A] The filter criteria for four transcription factor prediction databases:

JASPAR: The relative profile score threshold was 85%, and scores > 10.

hTFtarget: Default options.

KnockTF: Fold change > 1.5.

ChIPBase: Regulatory domain is [-5 kb, 1 kb] (motif: N).

[B] **Metascape**: Default parameters, $P < 0.001$.

Table. S2 The results of LiP-MS assay (58 peptides corresponding to 47 proteins).

Protein ID	Gene	Protein Description	LogFC	P value	sig	Differential Peptides
P00441	SOD1	Superoxide dismutase [Cu-Zn]	1.757941	0.039264	1	3
P04179	SOD2	Superoxide dismutase [Mn], mitochondrial	-2.18504	0.031462	-1	3
P13639	EEF2	Elongation factor 2	1.192139	0.010485	1	3
P05023	ATP1A1	Sodium/potassium-transporting ATPase subunit alpha-1	1.207645	0.040379	1	2
P05556	ITGB1	Integrin beta-1	-1.3916	0.029395	-1	2
P49327	FASN	Fatty acid synthase	1.350013	0.012567	1	2
P62805	H4C1	Histone H4	-1.70587	0.047864	-1	2
P99999	CYCS	Cytochrome c	1.216416	0.007796	1	2
O00469	PLOD2	Procollagen-lysine, 2-oxoglutarate 5-dioxygenase 2	-1.14951	0.034489	-1	1
O15372	EIF3H	Eukaryotic translation initiation factor 3 subunit H	-1.04881	0.002325	-1	1
O60814	H2BC12	Histone H2B type 1-K	-2.45322	0.046175	-1	1
P00403	MT-CO2	Cytochrome c oxidase subunit 2	1.005477	0.016238	1	1
P01889	HLA-B	HLA class I histocompatibility antigen, B alpha chain	1.31956	0.033705	1	1
P04004	VTN	Vitronectin	-1.31372	0.038486	-1	1
P04183	TK1	Thymidine kinase, cytosolic	-1.39406	0.022726	-1	1
P06744	GPI	Glucose-6-phosphate isomerase	-1.10411	0.042158	-1	1
P06748	NPM1	Nucleophosmin	1.605	0.037427	1	1
P07355	ANXA2	Annexin A2	-2.0903	0.035699	-1	1
P14618	PKM	Pyruvate kinase PKM	-1.16427	0.031092	-1	1

P16435	POR	NADPH-cytochrome P450 reductase	-1.30951	0.014312	-1	1
P17813	ENG	Endoglin	-1.5821	0.009999	-1	1
P20618	PSMB1	Proteasome subunit beta type-1	-1.41923	0.049924	-1	1
P22314	UBA1	Ubiquitin-like modifier-activating enzyme 1	1.093167	0.016195	1	1
P26006	ITGA3	Integrin alpha-3	1.561683	0.040939	1	1
P27635	RPL10	Large ribosomal subunit protein uL16	1.184193	0.006612	1	1
P29317	EPHA2	Ephrin type-A receptor 2	1.14719	0.016357	1	1
P35268	RPL22	Large ribosomal subunit protein eL22	1.412971	0.022976	1	1
P43490	NAMPT	Nicotinamide phosphoribosyltransferase	1.994798	0.000324	1	1
P51571	SSR4	Translocon-associated protein subunit delta	-1.23528	0.047331	-1	1
P53396	ACLY	ATP-citrate synthase	1.288085	0.045699	1	1
P55145	MANF	Mesencephalic astrocyte-derived neurotrophic factor	-1.21195	0.041896	-1	1
P60903	S100A10	Protein S100-A10	-1.21966	0.029774	-1	1
P61769	B2M	Beta-2-microglobulin	1.377048	0.016606	1	1
P62269	RPS18	Small ribosomal subunit protein uS13	-2.60091	0.039184	-1	1
P62316	SNRPD2	Small nuclear ribonucleoprotein Sm D2	-2.05467	0.038768	-1	1
P68032	ACTC1	Actin, alpha cardiac muscle 1	-1.17789	0.016415	-1	1
P80723	BASP1	Brain acid soluble protein 1	-1.47545	0.020415	-1	1
P83111	LACTB	Serine beta-lactamase-like protein LACTB, mitochondrial	2.429089	0.016153	1	1

Q01780	EXOSC10	Exosome component 10	-2.3035	0.047053	-1	1
Q02878	RPL6	Large ribosomal subunit protein eL6	1.838028	0.048915	1	1
Q13724	MOGS	Mannosyl-oligosaccharide glucosidase	1.166198	0.047663	1	1
Q14192	FHL2	Four and a half LIM domains protein 2	-1.65314	0.037883	-1	1
Q15393	SF3B3	Splicing factor 3B subunit 3	1.285144	0.002469	1	1
Q15942	ZYX	Zyxin	1.276395	0.031141	1	1
Q9NZM1	MYOF	Myoferlin	1.002151	5.22E-05	1	1
Q9UJZ1	STOML2	Stomatin-like protein 2, mitochondrial	-1.03778	0.039183	-1	1
Q9UK76	JPT1	Jupiter microtubule associated homolog 1	1.062238	0.045057	1	1

Table. S3 Reactome pathway analysis of the different peptides listed in Table S2.

Description	Gene Ratio	Counts	LogP	Log(q-value)	Symbols
Viral Infection Pathways	0.018252934	14	-11.20475564	-6.860225827	ATP1A1, B2M, HLA-B, ITGB1, NPM1, PSMB1, RPL6, RPL10, RPL22, RPL18, SNRPD2, MOGS, H4C9, H2BC12
**Cellular responses to stress**	0.01376721	11	-7.5314894	-3.664080816	COX2, PSMB1, RPL6, RPL10, RPL22, RPS18, SOD1, SOD2, H4C9, CYCS, H2BC12
SARS-CoV Infections	0.019323671	8	-6.64888503	-3.102228908	ATP1A1, B2M, HLA-B, ITGB1, NPM1, RPS18, SNRPD2, MOGS
Peptide chain elongation	0.055555556	5	-6.54130067	-3.102228908	EEF2, RPL6, RPL10, RPL22, RPS18
Translation	0.0239726	7	-6.48762663	-3.102228908	EEF2, RPL6, RPL10, RPL22, RPS18, SSR4, EIF3H

Table. S4 Baseline information of 5 enrolled ARDS patients.

No.	Gender	Age	Causes of ARDS	Other respiratory diseases	Exposure to COVID-19
Patient #1	Male	57	Pneumonia	No	Yes
Patient #2	Male	60	Pneumonia	Fungal pneumonia	No
Patient #3	Female	56	Pneumonia	No	No
Patient #4	Male	87	Pneumonia	Chronic obstructive pulmonary disease	No
Patient #5	Male	60	Interstitial pneumonia	No	No

Table. S5 Antibodies used in this study.

Antibody	Sources	Applications
ACCTUB	Santa Cruz (Cat#Sc-23950)	1:50 for IF
ACSL4	Proteintech (Cat#22401-1-AP)	1:1000 for WB
β-Tubulin	Cell Signaling Technology (Cat#2146)	1:1000 for WB
CREB1	Cell Signaling Technology (Cat#9197)	1:1000 for WB
FTH1	Abclonal (Cat#A19544)	1:1000 for WB
GAPDH	Cell Signaling Technology (Cat#5174)	1:1000 for WB
GPX4	Abcam (Cat#ab125066)	1:5000 for WB
HMOX1	Abcam (Cat#ab189491)	1:1000 for WB; 1:250 for IF; 1:10000 for IHC
JUN	Cell Signaling Technology (Cat#9165)	1:1000 for WB
MUC5AC	Absin (Cat#abs126767)	1:50 for IF
p-CREB1 (Ser133)	Cell Signaling Technology (Cat#9198)	1:500 for WB; 1:400 for IHC; 1:25 for ChIP
p-JUN (Ser73)	Cell Signaling Technology (Cat#3270)	1:1000 for WB
p-STAT3 (Tyr705)	Cell Signaling Technology (Cat#9145)	1:1000 for WB

SLC7A11	Abcam<br>(Cat#ab175186)	1:1000 for WB
SOD1	Proteintech<br>(Cat#10269-1-AP)	1:5000 for WB
STAT3	Cell Signaling Technology<br>(Cat#9139)	1:1000 for WB

Table. S6 Clinical characteristics of patient-derived hAOs.

No.	Gender	Age	Cancer type	TNM stage
Patient #1	Male	74	Lung squamous cell carcinoma	T3 N2 M0
Patient #2	Male	56	Lung adenocarcinoma	T4 N2 M0

Table. S7 Airway organoids medium recipes used in this study.

Medium component	Final concentration	Sources	Cat.
Advanced DMEM/F12	1×	Invitrogen	#12634-034
A8301	500 nM	Tocris	#2939
B27 supplement	1×	Gibco	#17504-44
EGF-7	25 ng/mL	Peprotech	#100-19
EGF-10	100 ng/mL	Peprotech	#100-26
N-Acetylcysteine	1.25 mM	Sigma	#A9165-5g
Nicotinamide	5 mM	Sigma	#N0636
Noggin	100 ng/mL	Peprotech	#120-10C
Primocin	50 μg/mL	Invitrogen	#Ant-pm-1
R-Spondin	500 ng/mL	Peprotech	#120-38
SB202190	500 nM	Sigma	#S7067
Y-27632	5 μM	Abmole	#M20999

Table. S8 Targeting sequences for siRNAs, shRNAs, and sgRNA used in this study.

Notes	Sequences (5'-3')
siJUN^{-1}	GGATCAAGGCGGAGAGGAA
siJUN^{-2}	CCAAGAACGTGACAGATGA
siCREB1^{-1}	GCCACAGATTGCCACATTA
siCREB1^{-2}	GACACATGATGGAGATGAT
shHMOX1^{-1}	GCTGAGTTCATGAGGAACTT
shHMOX1^{-2}	CTGTAGGGCTTTATGCCATGT
shSOD1^{-1}	CAATGTGACTGCTGACAAA
shSOD1^{-2}	GTTCATGAGTTTGGAGATA
sgCREB1	GCTCGAGAGTGTCGTAGAA
shSc / siCtrl	TTCTCCGAACGTGTCACGT

Table. S9 Primer sequences used for qRT-PCR in this study.

Notes	Primer sequences (5'-3')
hHMOX1-F	AAGACTGCGTTCCTGCTCAAC
hHMOX1-R	AAAGCCCTACAGCAACTGTCG
hAGER-F	GTGTCCTTCCCAACGGCTC
hAGER-R	ATTGCCTGGCACCGGAAAA
hSFTPD-F	AAGCAGGGGAACATAGGACCT
hSFTPD-R	ACACCTCGCTCTCCCTTAGG
hICAM1-F	ATGCCCAGACATCTGTGTCC
hICAM1-R	GGGGTCTCTATGCCCAACAA
hvWF-F	CCGATGCAGCCTTTTCGGA
hvWF-R	TCCCCAAGATACACGGAGAGG
hGAPDH-F	CTGGGCTACACTGAGCACC
hGAPDH-R	AAGTGGTCGTTGAGGGCAATG

Table. S10 The primers used to construct the reporter gene and dual-luciferase reporter assay.

Notes	Forward primers (5'-3')	Reverse primers (5'-3')
Wild-type	GAAGATCTTCCCTGAATGTGCCTGGAAGAGTGTC	GGGGTACCCCGGGCTCGTTCGTGCTGGC
Mutated	CTTCCTGCACCCCTCTGAGCCTC	AGTCCACATAAAGCAGGATGCCAG

Table S11. Primers used for the ChIP assays in this study.

Notes	Sense (5'-3')	Antisense (5'-3')
Site #1 (PCR)	AGGGCAGCTTTAATGGTAGGCA	GGGAGATGCCATGCTGCTAG
Site #2 (PCR)	GGGGTGTTTTGGCATCAGAAG	GCAAGGAAGACAAGCCCAAGTTA
Site #3 (PCR)	CGCTCAGGCCTGTCATCCCA	CGTGGGTGTGGGTTAGGGTGG
NBR (PCR)	AGCCACGTTTTCCCCATCTG	TCCCCACGCTGATGTTTTAAC
Site #1 (qPCR)	CTGCCTCATCTCTTCTGCCTCCA	CTGGGCACGGTGGCTCATACC
Site #2 (qPCR)	AAGCGATTCTCCTGCCTCAGCT	AGCACTCTGGGAGCTGAGGTGG
Site #3 (qPCR)	TCGCTCAGGCCTGTCATCCC	ACGCAATTCTCCTACCTCAGCCTC
